# Supplementary material for: The role of elasticity on adhesion and clustering of neurons on soft surfaces
Source: Commun Biol. 2024 May 23;7:617. doi: 10.1038/s42003-024-06329-9 (PMC11111731; doi:10.1038/s42003-024-06329-9)
Supplement: Supplementary file 2 — Supplementary Information [file 42003_2024_6329_MOESM2_ESM.pdf]

The role of elasticity on adhesion and clustering of neurons on soft surfaces

Giovanni Marinaro[1], Luigi Bruno[2], Noemi Pirillo[3], Maria-Laura Coluccio[3], Marina Nanni[4], Natalia Malara[5], Edmondo Battista[6], Giulia Bruno[7], Francesco De Angelis[7], Laura Cancedda[4], Daniele Di Mascolo[8, 9, *], Francesco Gentile[3, *]

[1] Center for Interdisciplinary Research on Medicines (CIRM), Quartier Hôpital, 4000 Liège, Belgium

[2] Department of Mechanical Engineering, Energy Engineering and Management
University of Calabria, 87036 Rende (Italy)

[3] Nanotechnology Research Center, Department of Experimental and Clinical Medicine
University of "Magna Graecia" of Catanzaro, 88100 Catanzaro, Italy

[4] Department of the Neuroscience and Brain Technology, Italian Institute of Technology
Via Morego 30, 16163 Genoa, Italy

[5] Department of Health Science, University of "Magna Graecia", 88100 Catanzaro, Italy

[6] Department of Innovative Technologies in Medicine & Dentistry, University "G. d'Annunzio" Chieti-Pescara, 66100, Chieti, Italy

[7] Plasmon Nanotechnologies, Italian Institute of Technology
Via Morego 30, 16163 Genoa, Italy

[8] Laboratory of Nanotechnology for Precision medicine, Italian Institute of Technology
Via Morego 30, 16163 Genoa, Italy

[9] Department of Electrical and Information Engineering, Polytechnic University of Bari
70126 Bari, Italy

* Corresponding authors: daniele.dimascolo@poliba.it, francesco.gentile@unicz.it

Supplementary Information

#	Supplementary Note
1	*Mechanical characterization of soft PDMS surfaces*
2	*Scanning Electron Microscopy analysis of PDMS surfaces*
3	*Power spectrum of soft PDMS surfaces*
4	*Characterization and analysis of the chemical structure and composition of PDMS sample-surfaces, and the interaction between PDMS and poly-d-lysine, by Raman spectroscopy and Energy Dispersive X-Ray Analysis (EDAX).*
5	*Fluorescence images of cells cultured on soft PDMS surfaces*
6	*Neuronal networks determined from the fluorescence images of cells on soft PDMS surfaces*
7	*SW characteristics of neuronal cell graphs for different values of the cut-off probability p – a parameter in the cells-wiring model*
8	*Direct reconstruction and characterization of neuronal cell graphs by neuronal branching analysis from green fluorescent images*
9	*Characterization of PDMS leakage into DI water*
10	*A mathematical model of adhesion and clustering on soft surfaces*
11	*Colocalization analysis between neurons and the overall cell population on conventional flat surfaces*
12	*Methods: determining the fractal dimension of PDMS surfaces*
13	*Methods: cell image analysis and processing*
14	*Methods: neuronal cells wiring*
15	*Methods: simulating information flows in neuronal cell networks*
16	*Methods: functional multi calcium imaging*

Supplementary Note 1. *Mechanical characterization of soft PDMS surfaces*.

The mechanical characterization of the PDMS was carried out by both tensile and nano-indentation tests. The first type of test has the advantage of providing highly reliable results, independent from the shape and the dimensions of the specimen; on the other hand the tensile test cannot be carried out on prototypes or components in-situ, but is destructive and has to be performed on specimens properly and accurately prepared. Alternatively, an instrumented indentation test is non- or semi-destructive, does not require specific specimen preparation except of a local surface treatment, but the numerical values obtained for the mechanical properties could be strongly dependent on testing conditions.

With the aim of exploiting the strengths of both approaches, the PDMS, produced at different percentage of siloxane ratios, was initially tested by tensile tests carried out on thin strips of material of uniform width and thickness. Afterward, the same materials, shaped as thick disk specimen whose one of the two faces was properly grinded and polished, were tested by an instrumented nano-indentation test, and a correlation equation was evaluated between the mechanical properties obtained by the two approaches.

Tensile test

The tensile tests were carried out according to the procedure adopted in (*1*), whose schematic of the experimental setup is shown in the **Supplementary Figure 1.1**. The specimens, produced as strips 5 mm wide and 2 mm thick, were tested by the universal testing machine MTS model Criterion 42, consisting of a single column tabletop-framed electromechanical testing system, equipped with the load cell LSB.102 (load capacity 100 N, class 0.5 from 1% to 100% of load capacity). The load was applied by manual vice action grips for testing paper, plastic film, textiles, sheet materials and packaging components. The initial distance between the grips at the beginning of each test was set to 20 mm.

All the specimens were tested in displacement control mode, by setting the speed of the moving cross head to 2 mm/min. The single test terminates with the failure of the specimen or when the length of the specimen is about doubled, whichever event occurs before.

Voltages generated by the universal testing machine proportional to the load and displacement values were used as reference signals to synchronize the images acquired by the camera, a Prosilica

model ATV-GT2450 (resolution 2448 × 2050 pixel, pixel dimension 3.45 μm × 3.45 μm, maximum frame rate 15 Hz). In particular, the acquisition software (VIC-Snap, Correlated Solutions) allows to set a time delay and/or the increment of one of the synchronization signals to acquire the sequence of images. In the experiments carried out in the present work, these reference values were set to 1 s (maximum time delay) and 50 mm (displacement increment of the moving cross head). The image illumination was optimized by two diode light sources placed symmetrically oriented with respect to the normal to the specimen surface (*z*-axis). All the signals (voltages and images) were acquired by a workstation interfaced with all devices by way of the data acquisition system ISI-SYS model DAQ-STD-8D.

The software VIC-2D (Correlated Solutions) was used to evaluate the in-plane displacement components. A Region Of Interest (ROI) of about 3 mm × 3 mm far from the clamping area and from the edges of the specimen was analyzed with the correlation algorithm, by setting a subset of 21 x 21 pixel and a step of 5 pixel in both horizontal and vertical directions. The magnification ratio resulted to be about 11 mm/pixel, the two displacement components were evaluated on about 3000 points over the ROI. The strain field was calculated by differentiating the displacement field, fitted on the ROI by bilinear functions. **Supplementary Figure 1.2** shows in five different loading configurations the distribution of the axial component (along y axis) of the displacement vector plotted on the images of the specimen used to run the DIC analysis, a video showing the whole test is linked to the figure.

Nano-indentation test

The instrumented indentation tests were carried out according to the procedure introduced by Oliver and Pharr (*2*), whose success brought the drafting and the release of different standards (*3*, *4*), and several research papers (*5*, *6*). Notice though that, along with the Oliver & Pharr's, also the Hertzian model can be used to explain and interpret AFM nanoindentation experiments on biological samples, as evidenced by previously reported works (*7*, *8*).

In the specific case of the present study, the Young's modulus measured by the indentation test was correlated with the results obtained by a conventional tensile. This mechanical property, calculated as the slope of the initial portion of the stress-strain curve recorded during the tensile

test, can be evaluated by a standard instrumented indentation test consisting of three phases: loading, pause and unloading.

Figure 3 shows the typical experimental data set obtained by an instrumented indentation test carried out in load control. The penetration depth (*d*) and the load (P) are registered at a specific sampling rate, by the temporal distribution of the *d* (**Supplementary Figure 1.3a**) and P (**Supplementary Figure 1.3b**) the overall curve is attained as in the **Supplementary Figure 1.3c**, by which the Young's modulus and other properties can be evaluated. Young's modulus, according to the aforementioned theory, is proportional to the initial slope of the unloading curve S_U, and is calculated by a specific procedure depending on the operating conditions of the test.

The indentation tests were performed by an Anthon-Parr instrumented hardness station, available at the lab of Material Characterization (LCM) of STAR-Lab at the University of Calabria. This specific equipment is configurated into three modules: the nano-indenter, the micro-indenter, and the optical microscope. In the present study, only the nano-indenter and optical microscope modules were used. In order to define a protocol to measure the Young's modulus the following parameters has to be defined.

- *Testing scale and indenter type*. The surface structure could affect significantly the measurements due to the small loads and displacements occurring on the contact area. In addition, the properties' entity retrieved by this kind of approach can change considerably according to the amount of material involved in the mechanical test. Hence it is necessary to establish either to use the nano- or a micro-indentation test head and which kind of indenter to mount in it (e.g. Vickers, Berkovich, corner cube, spherical).
- *Control variable and peak value*. The indentation test can be carried out in load or displacement control mode, depending which parameter is controlled according to a specific temporal law. In addition, one or more reference values are assigned to the control parameter in order to define the beginning and the end of the test.
- *Loading and unloading rate*. The temporal distribution of the control variable could be an important parameter, especially for those materials exhibiting a viscous mechanical behavior. This type of setting, in fact, could impact significantly the values of mechanical properties measured by an instrumented indentation test: some mechanical properties are strain rate dependent, as well as a the higher the strain rate the lower the impact of viscous

phenomena. In the specific case of a standard test, as depicted in the **Supplementary Figure.3b**, the loading $\dot{P}_L$ and unloading $\dot{P}_U$ rates are required, consisting in two speed values to assign to the control variable.

– *Pause duration.* In order to qualitatively estimate the potential viscous behavior of the material and to give the material enough time for strain settlement, one or more pause steps can be assigned to the control variable during the test. In the specific case of a standard test, as depicted in the **Supplementary Figure 1.3b**, a single pause Dt is necessary between loading and unloading ramps.

After several test carried out on the material subject of the present study, the parameters defining the measurement protocol were chosen and reported in the **Supplementary Table 1**. Before performing the indentation tests, the specimens' surface were ground and polished by a Struers grinding and polishing equipment, model Tegramin-25, using the grinding/polishing protocol prescribed for soft polymers. It is a four-step protocol requiring the use of SiC foils with higher grit number: 500, 1000, 2000, and 4000. In all the surface finishing operation water was used as lubricant, a force of 15 N was applied between the foil and the specimen, and the rotation speed of the tool was set to 150 rpm.

**Parameter**	**Value**	**Unit**
Head	Nano-indentation	-
Indenter	Berkovich	-
Indenter material	Diamond	-
Young's modulus	1141	GPa
Poisson's ratio	0.07	-
Control mode	Load-control	-
Peak value	60	mN
Loading rate	1	mN/s
Unloading rate	1	mN/s
Pause duration	50	s

Supplementary Table 1. Operative parameters chosen to defining the measurement protocol adopted in the present study.

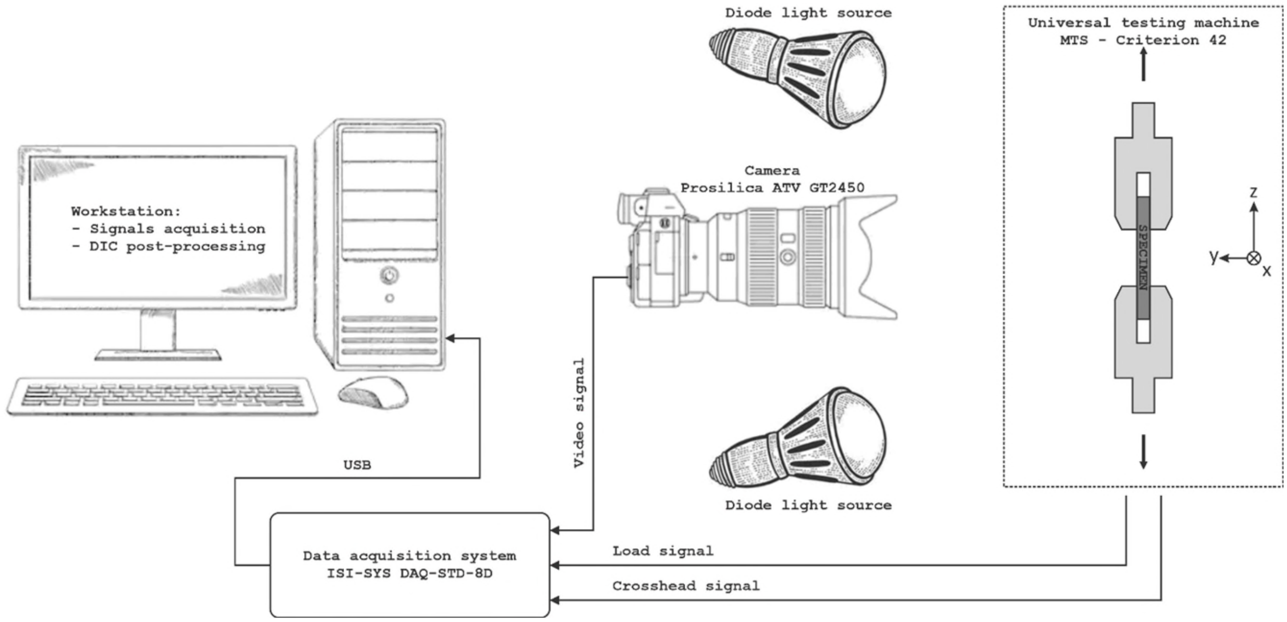

Supplementary Figure 1.1. Schematic of the experimental setup to apply a tensile test on a thin layer specimen whose strain field was measured by the full-field optical technique based on DIC methods.

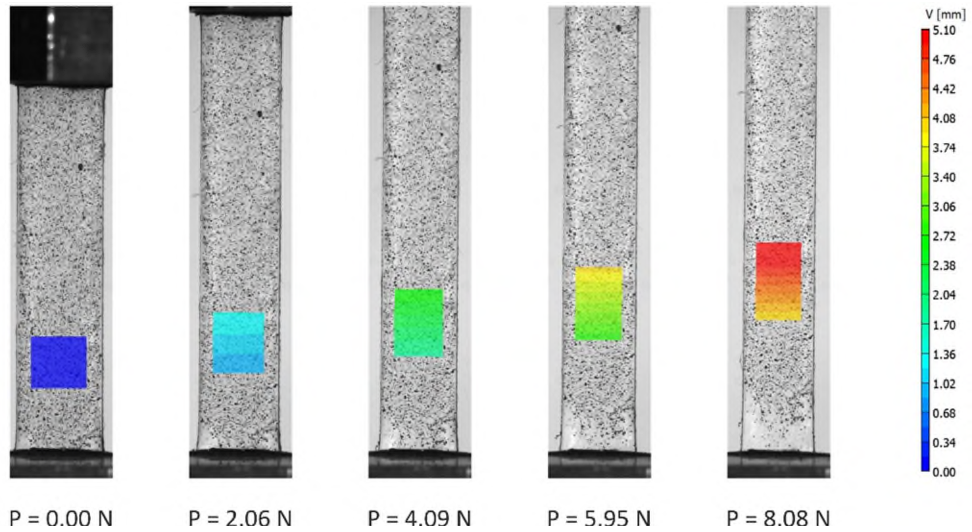

Supplementary Figure 1.2. Axial displacement field (V component, along y direction) evaluated by DIC method at 5 different loading configurations.

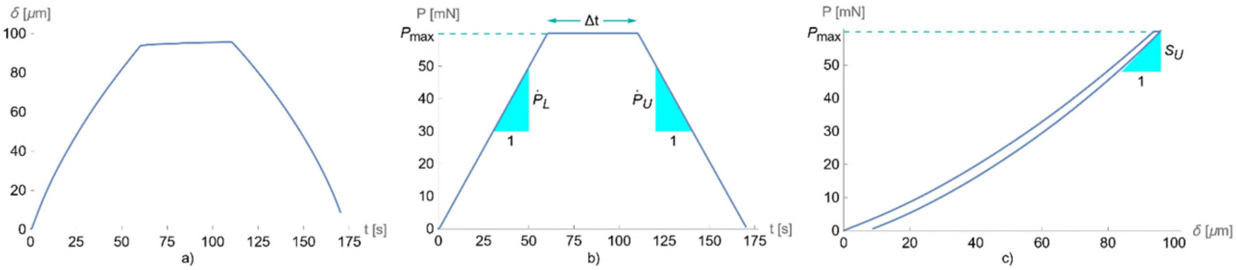

Supplementary Figure 1.3. Experimental data obtained from the instrumented indentation test: a) penetration depth vs time; b) load vs time; c) load vs penetration depth.

Supplementary Note 2. *Scanning Electron Microscopy analysis of PDMS surfaces*.

We performed Scanning Electron Microscopy (SEM) analysis of PDMS sample surfaces. The analysis was performed using a dual beam Helios Nanolab 600 (Thermo Fisher) scanning electron microscope. Samples were sputtered with $10\ nm$ of gold before imaging and glued to a standard focused ion beam/SEM stub with silver glue. The images were carried out with the sample tilted at $45°$ in immersion mode, and the back-scattered electrons were collected with a current of $0.20\ nA$ and a $3\ kV$ acceleration of the electronic beam. **Supplementary Figure 2.1** illustrates the morphology of a sample surface - for a polymeric-base:curing agent ratio of 14 and relative value of elastic modulus of $1\ MPa$. The morphology of PDMS measured by SEM is consistent with the values of surface roughness determined by laser interferometry of about $20\ nm$.

The same SEM analysis was performed on PDMS sample surfaces with values of elasticity of $0.55,\ 1.88,\ 2.65\ MPa$ (**Supplementary Figure 2.2**). In all considered examples, surface profile is never perfectly flat. Instead, it always exhibits some spatial variability. Surface texture is consistent with morphology measurement performed by quantitative interferometry analysis.

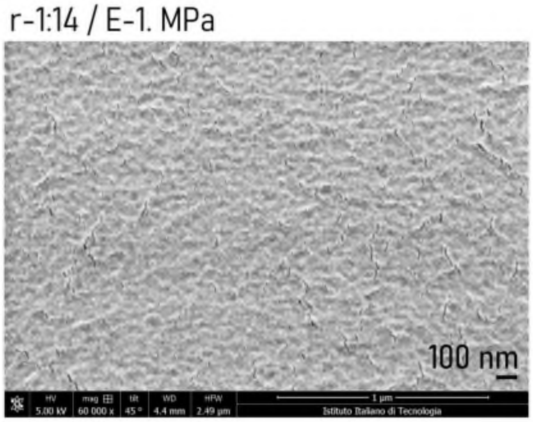

Supplementary Figure 2.1. PDMS surface morphology examined by scanning electron microscopy for a sample preparation corresponding to a value of elasticity of 1 MPa.

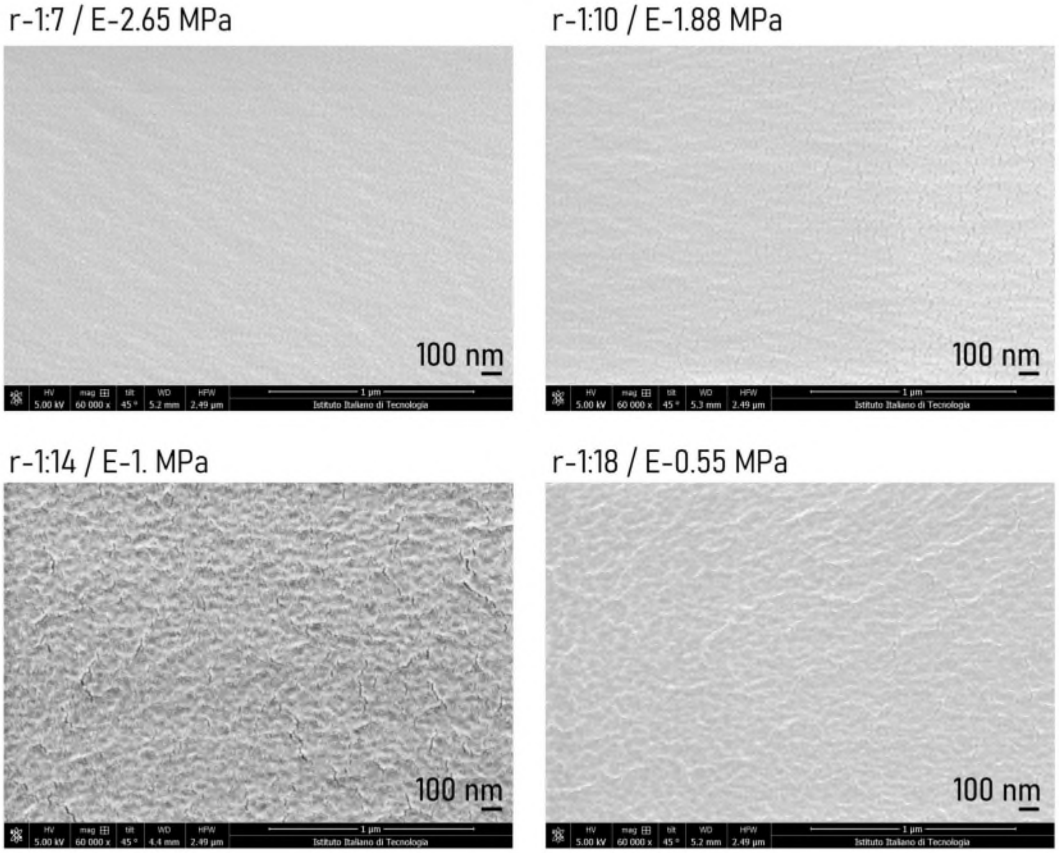

Supplementary Figure 2.1. PDMS surface morphology examined by scanning electron microscopy for a sample preparation corresponding to a values of elasticity spanning the $0.55 - 2.65$ MPa range.

Supplementary Note 3. *Power spectrum of soft PDMS surfaces*.

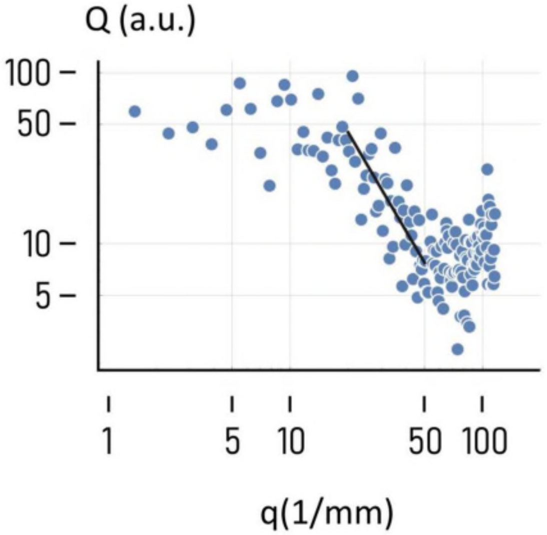

Supplementary Figure 3.1. Power Spectrum density function of the topography of PDMS soft surface measured by laser interferometry and reported in ***Figures 2.d*** and ***e*** of the main article.

Supplementary Note 4. *Characterization and analysis of the chemical structure and composition of PDMS sample-surfaces, and the interaction between PDMS and poly-d-lysine, by Raman spectroscopy and Energy Dispersive X-Ray Analysis (EDAX).*

Raman spectra of PDMS samples were obtained using a Renishaw InVia Microscope with a 1024 CCD detector, an excitation wavelength of 633 nm, and 50 × objective lens. The laser power was maintained at a constant value of 300 mW and the integration time was set to 50 s throughout all measurements. We acquired Raman spectra of samples at different proportions ρ of the SYLGARD 184 polymeric base to the curing agent. Values of ρ were varied from 7: 1 to 18: 1 - the elasticity of resulting samples changed accordingly from ∼2.65 to ∼0.55 MPa. To assure statistical significance, for each sample we performed 7 × 7 measurements over a square region of 70 $\mu m \times 70\ \mu m$. After acquisition, Raman spectra were base line corrected to remove background. Then, we performed min-max normalization and cosmic rays removal (*1*). Remarkably, resulting spectra (**Supplementary Figure 4.1**) exhibit peaks typical of polydimethylsiloxane (*9*, *10*), and specifically at: 491.8, 615.6, 707.6, 786.0, 847.7, 1242.8, 1393.3 cm^{-1}. That correspond to the following vibrational modes: Si–O–Si stretching (491.8 cm^{-1}); Si–C asymmetrical (615.6 cm^{-1}), symmetrical (707.6 cm^{-1}) and combined (786.0 cm^{-1}) stretching; CH3 asymmetrical stretching (847.7 cm^{-1}) and asymmetrical bending (1393.3 cm^{-1}); SiCH3 symmetrical bending (1242.8 cm^{-1}).

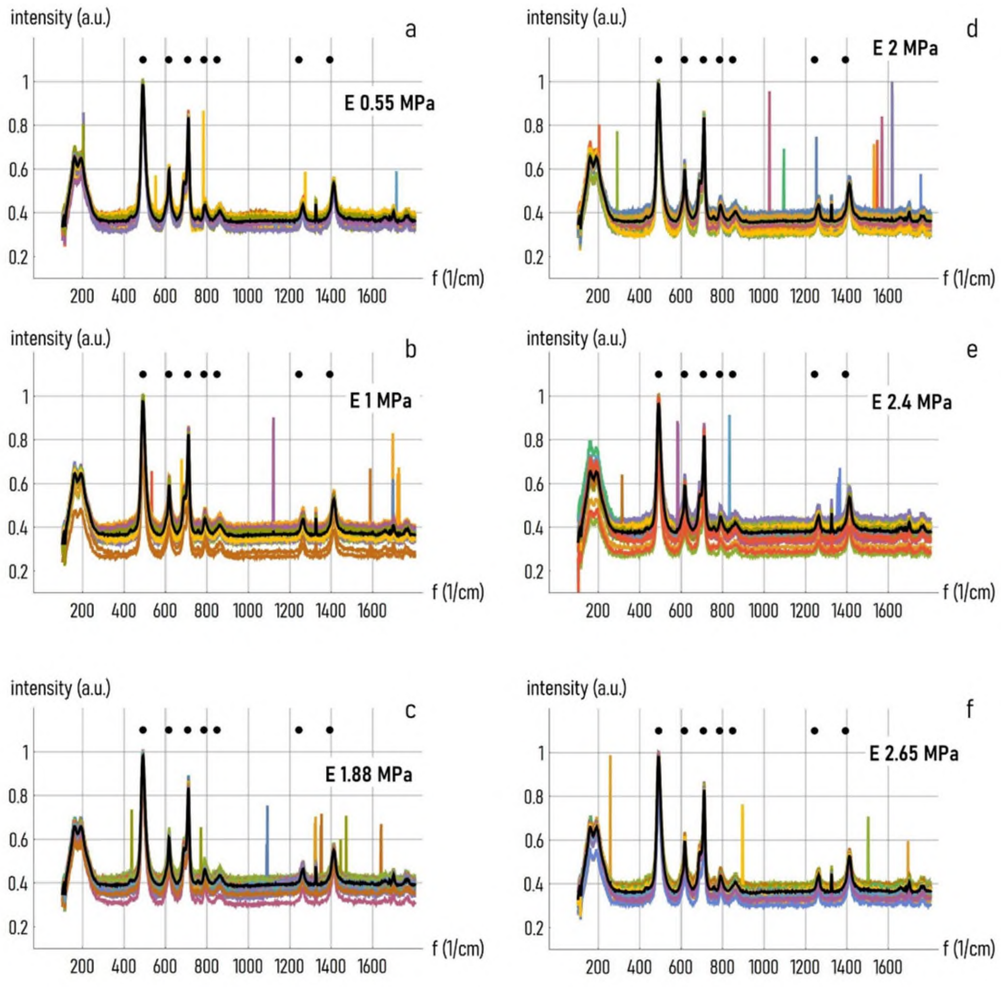

Supplementary Figure 4.1. Raman spectrum and average Raman spectrum of PDMS surfaces for different material elasticity values.

Supplementary Figure 4.1 illustrates Raman spectra of PDMS samples for different sample-preparations. For each value of surface elasticity, Raman spectra of the same sample are reported in the same diagram. In the diagrams, the black, thick-line graph is the average spectrum. Visual, *qualitative* inspection of graphs suggests that there are minimal differences between samples. To measure *quantitatively* how the Raman signature of PDMS samples varies as a function of sample preparation, we report in the **Supplementary Figure 4.2** the mean-peak intensity as a function of surface elasticity, evaluated for values frequency typical of PDMS, i.e. 491.8, 615.6, 707.6, 786.0, 847.7, 1242.8, 1393.3 cm^{-1}. Peak-intensity variations are moderate for all considered frequencies, with an overall variance percentage comprised between 2% and 11% (**Supplementary Figure 4.2h**). This suggests that the chemical surface-structure of samples is

subject to minimal-important changes as substrate elasticity varies from 0.55 to 2.65 MPa - and the proportion ρ between the SYLGARD 184 polymeric base and the curing agent varies from 7:1 to 18:1.

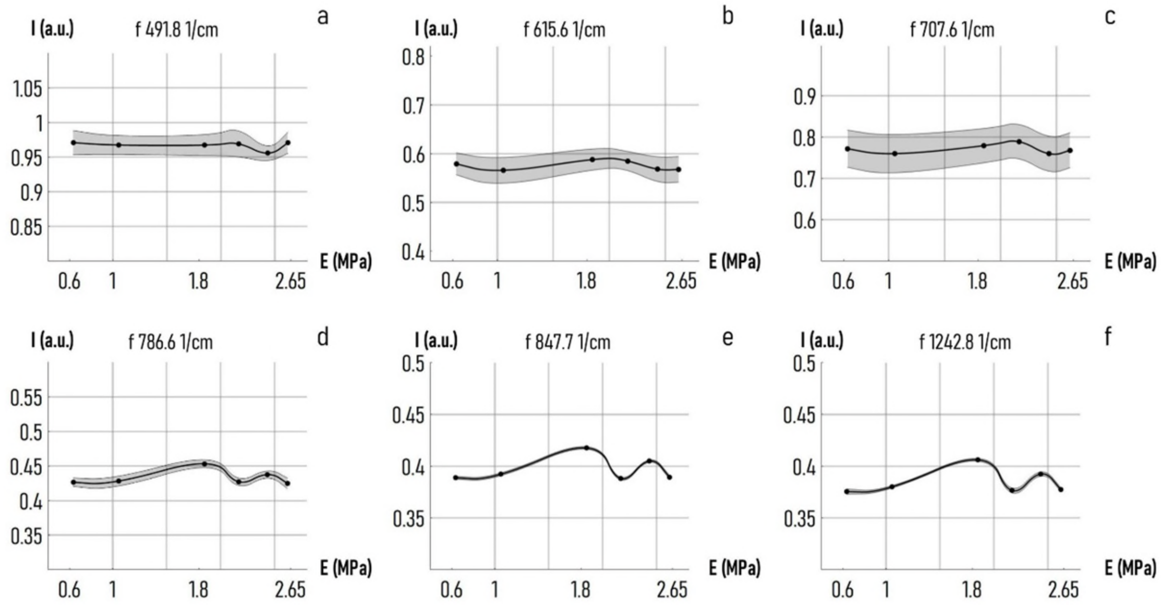

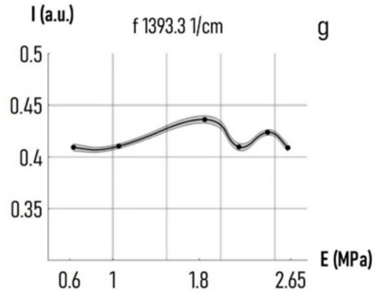

h

f (1/cm)	E (MPa) 0.55	1	1.88	2	2.4	2.65	max var
491.8	.97	.96	.97	.97	.95	.97	2%
615.6	.58	.56	.59	.58	.56	.57	5%
707.6	.77	.76	.78	.79	.76	.76	2.6%
786.6	.42	.43	.45	.43	.44	.42	7%
847.7	.39	.39	.42	.38	.40	.39	10%
1242.8	.37	.38	.41	.37	.39	.37	11%
1393.3	.41	.41	.44	.41	.42	.40	5%

Supplementary Figure 4.2. Raman intensity measured in correspondence of frequencies characteristic of PDMS as a function material elasticity (*a*-*g*). Numerical values of mean Raman intensities measured at different central frequencies, for different values of PDMS elasticity (*h*).

We further analyzed Raman spectra using principal components analysis (PCA). PCA is a multivariate technique of analysis that can reduce the dimensionality of a dataset by finding the variables that contribute to its variance (*11*), i.e. the principal components (PCs).

Raman spectra relative to all PDMS sample preparations were grouped in a single dataset and processed. After the analysis, we found that the first three components - PC1, PC2 and PC3 -

account for more than the 99% of the information content of the original data, thus we used PC1, PC2 and PC3 to represent the spectra in the following of the study. Scatter plots of PC1 vs PC2 (**Supplementary Figure 4.3a**), of PC2 vs PC3 (**Supplementary Figure 4.3b**) and of PC1 vs PC3 (**Supplementary Figure 4.3c**) indicate that data points are homogeneously distributed in the space of the principal components – without a clear clustering of points into separate groups. This in turn indicates that there are not statistically significant differences among Raman spectra associated to different sample preparations. This is also evidenced by the decision graph (**Supplementary Figure 4.3d**) relative to the data points in the PC space. The decision graph is a plot of the *density* against the *minimal distance to other points with greater density* of points in a metric space (*12*). In a similar graph, points that stand out from the other with high values of *density* and relatively high values of *distance* mark the cluster centers (*12*). Lack of outliers in the diagram in the **Supplementary Figure 4.3d** hints that there is not a clear distinction between sample groups – and that different sample preparations cannot be segregated by Raman analysis.

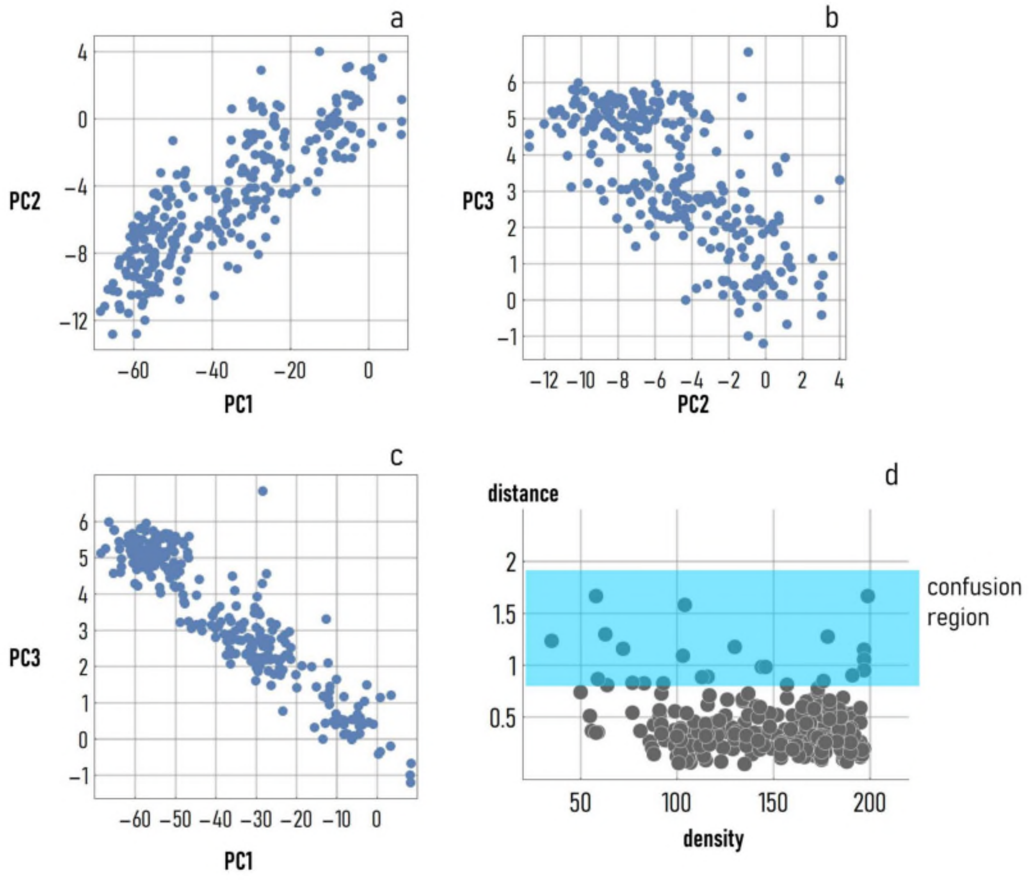

Supplementary Figure 4.3. PCA analysis of PDMS Raman spectra. Scatter plot of PC1 vs PC2 (*a*), PC2 vs PC3 (*b*) and PC1 vs PC3 (*c*) relative to the Raman spectra of PDMS for different values of material elasticity varying from 0.55 to 2.65 MPa. Decision graph relative to the principal components associated to Raman spectra of PDMS surfaces for different values of material elasticity varying from 0.55 to 2.65 MPa (*d*).

We then performed an additional Raman analysis on samples coated with poly-d-lysine (PD) to examine whether different PDMS sample preparations can affect PDMS-PD interaction. Since Raman peaks characteristic of PD fall in the $800 - 1800\ 1/cm$ range (*13, 14*), the measurement range was set to this interval. Raman spectra of PD-coated PDMS - reported in the **Supplementary Figure 4.4a** for the sole $E = 2$ MPa substrate - illustrate that the peaks typical of the components of PD are vanishingly small, perhaps sheltered by PDMS. With the remarkable exception of the peak at $1276\ 1/cm$, a hallmark of the amide III band of α-helix. Diagrams in the **Supplementary Figure 4.4b** show how the mean Raman spectrum of PD-coated-samples changes as a function of PDMS elasticity. The Raman intensity measured at $1276\ 1/cm$ is reported in the diagram of **Supplementary Figure 4.4c** as a function of material elasticity. Values of Raman intensity vary from 0.06 for $E = 0.55\ MPa$, to 0.064 for $E = 2.65\ MPa$. In the considered elasticity interval, the maximum variation of intensity is of $e \sim 36\%$, more relevant than the variations relative to the sole PDMS, but still less relevant than the variation in Young's modulus, that is of $\sim 380\%$.

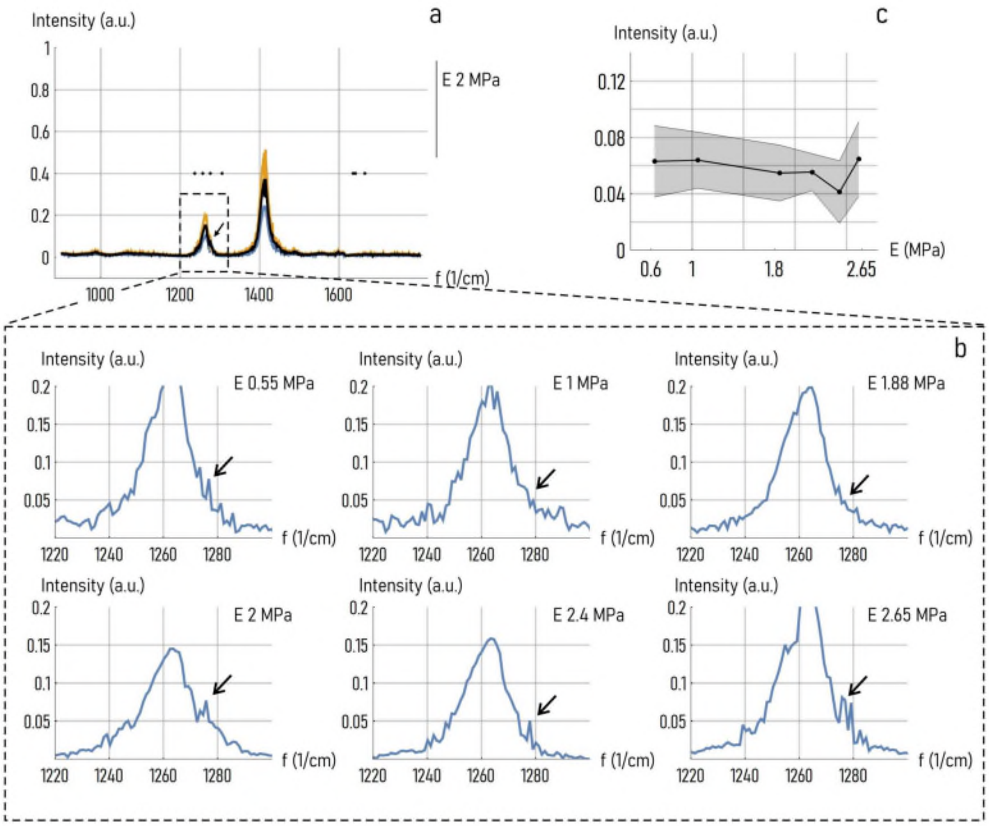

Supplementary Figure 4.4. Raman spectra and average Raman spectrum of PD-coated PDMS for value of Young's modulus E=2 MPa (*a*). Average values of Raman spectrum of PD-coated PDMS for different values of PDMS elasticity varying from 0.55 to 2.65 MPa (*b*). Trend of Raman intensity measured at 1276 1/cm as a function of the material Young's modulus (*c*).

Principal Components Analysis of Raman spectra indicates that the 1st, 2nd and 3rd principal components of Raman spectra do not seemingly cluster into groups (**Supplementary Figure 4.5a-c**), as illustrated by the decision graph relative to these data points the **Supplementary Figure 4.5d**. Thus Raman analysis and PCA processing of data suggest that the interaction between poly-d-lysine and PDMS is only marginally influenced by PDMS preparation.

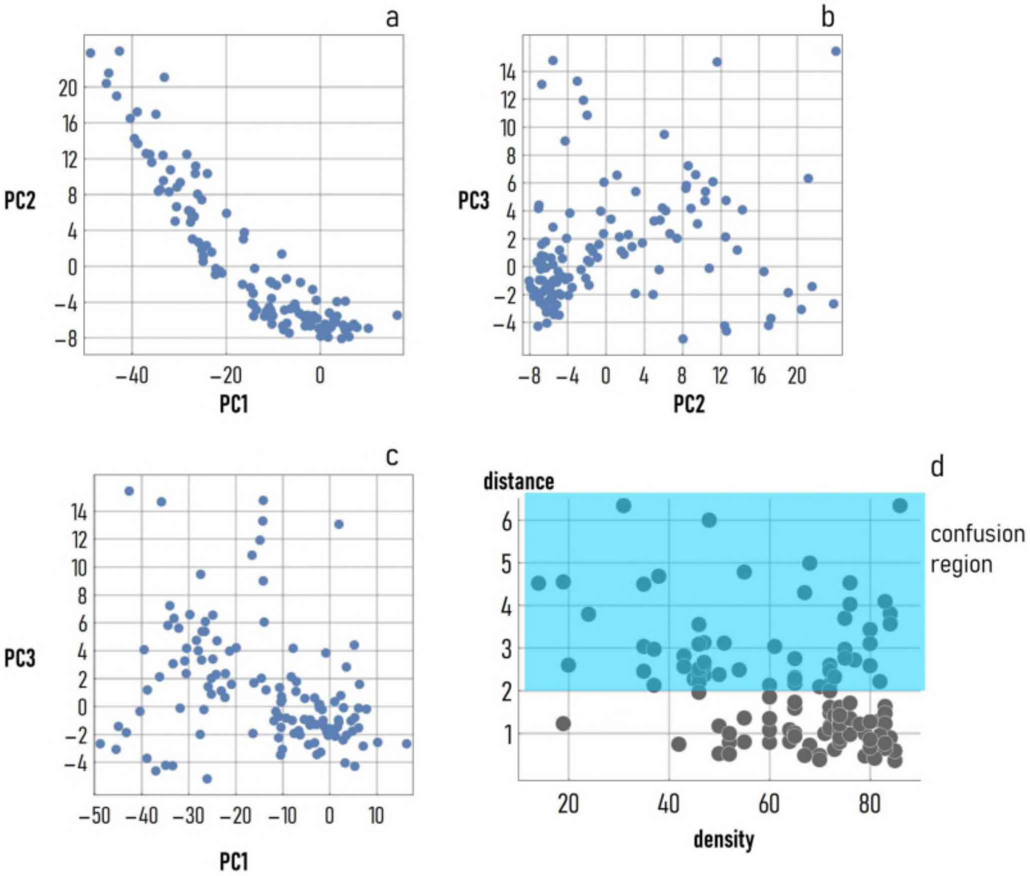

Supplementary Figure 4.5. PCA analysis of PD-coated PDMS Raman spectra. Scatter plot of PC1 vs PC2 (*a*), PC2 vs PC3 (*b*) and PC1 vs PC3 (*c*) relative to the Raman spectra of PDMS coated with PD for different values of material elasticity, varying from E-0.55 to E-2.65 MPa. Decision graph relative to the principal components associated to Raman spectra of PDMS surfaces coated with PD, for different values of material elasticity varying from 0.55 to 2.65 MPa (*d*).

To the end to characterize with maximum precision the chemical composition of PDMS samples, we further performed Energy dispersive X-ray analysis (EDAX) of samples. We performed EDAX on PDMS samples with a polymeric-base:curing-agent ratio varying between 7:1 and 18:1 – and Young's modulus spanning the $0.55 - 2.65$ MPa range. We used for the analysis a dual beam Helios Nanolab 600 (Thermo Fisher) scanning electron microscope. Samples were mounted on a standard stub and analyzed with a probe current of 0.20 nA and 5kV acceleration of the electronic beam. Results reported in the **Supplementary Table 2** illustrate that the composition of carbon (C), oxygen (O) and silicon (O) varies only marginally for different substrate preparations.

Sample #	ρ	E (MPa)	C (weight %)	O (weight %)	Si (weight %)
1	7	2.65	52.35	44.99	2.66
2	8	2.5	51.62	44.9	3.39
3	9	2	51.59	44.76	3.65
40	10	1.88	48.45	44.86	6.73
5	14	1	51.56	44.76	3.34
6	18	0.55	50.02	44.87	5.11

Supplementary Table 2. Elemental analysis of PDMS samples.

Overall, results presented in this section and conveniently reported in a separate supporting information, suggest that the polymeric-base:curing-agent ratio ρ influences significantly the Young's modulus of PDMS samples, and affects marginally the chemical composition and structure of samples.

Supplementary Note 5. *Fluorescence images of cells cultured on soft PDMS surfaces.*

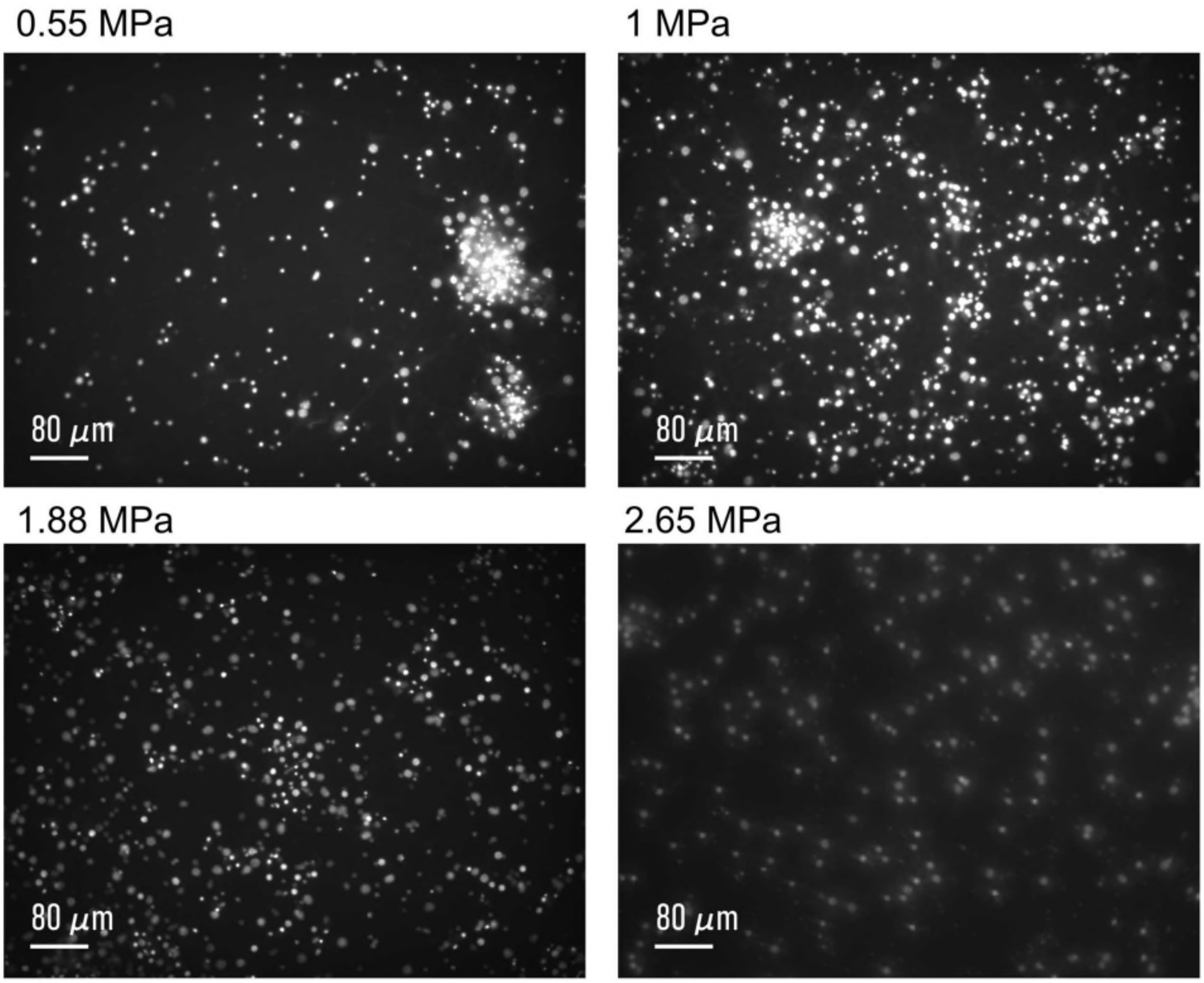

Supplementary Figure 5.1. Selection of fluorescence images of neuronal cells on PDMS surfaces taken $24h$ from culture for different values of substrate elasticity. In acquiring the images, cell-nuclei were selectively stained with DAPI, here visualized in grayscale colors.

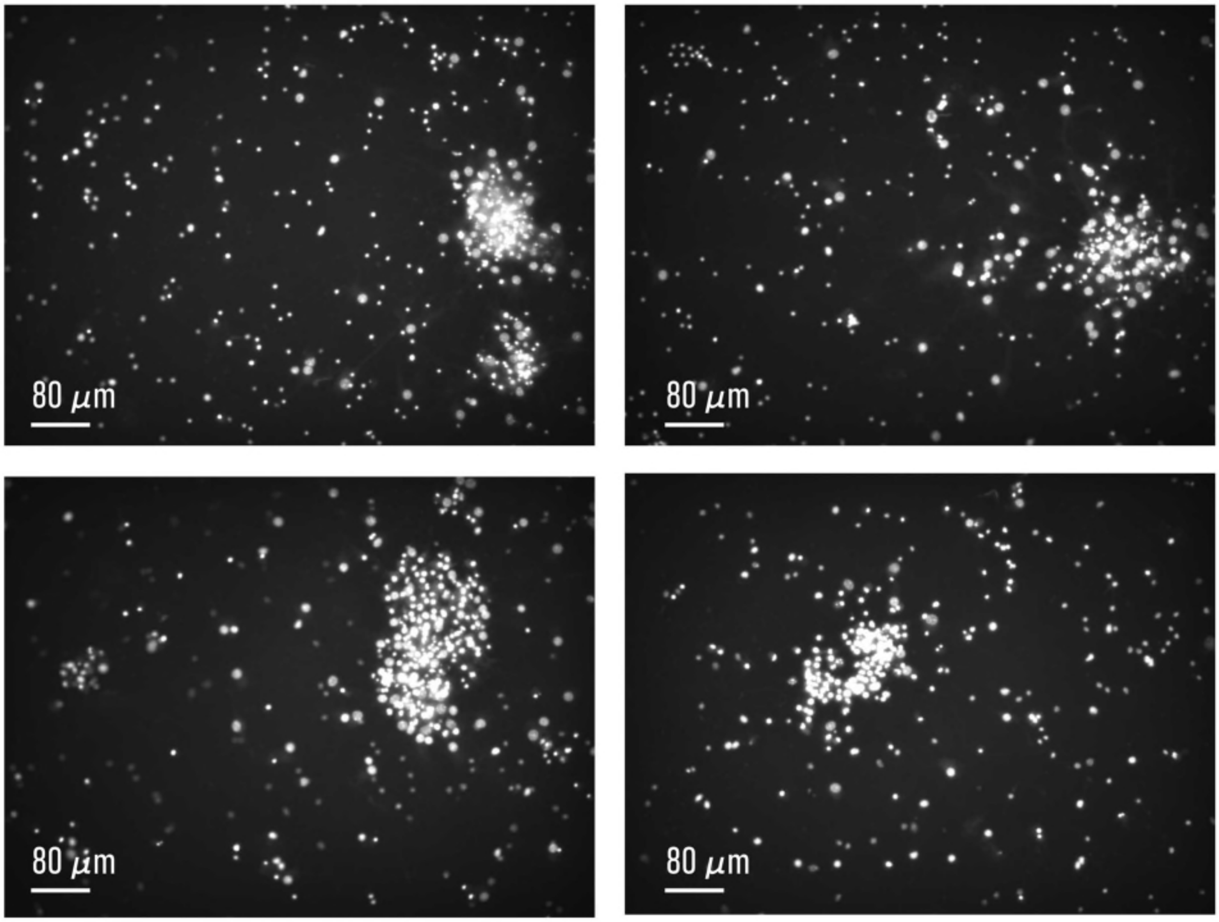

Supplementary Figure 5.2. Selection of fluorescence images of neuronal cells on PDMS surfaces taken $24h$ from culture. In acquiring the images, cell-nuclei were selectively stained with DAPI, here visualized in grayscale colors. ($E = 0.55\ MPa$).

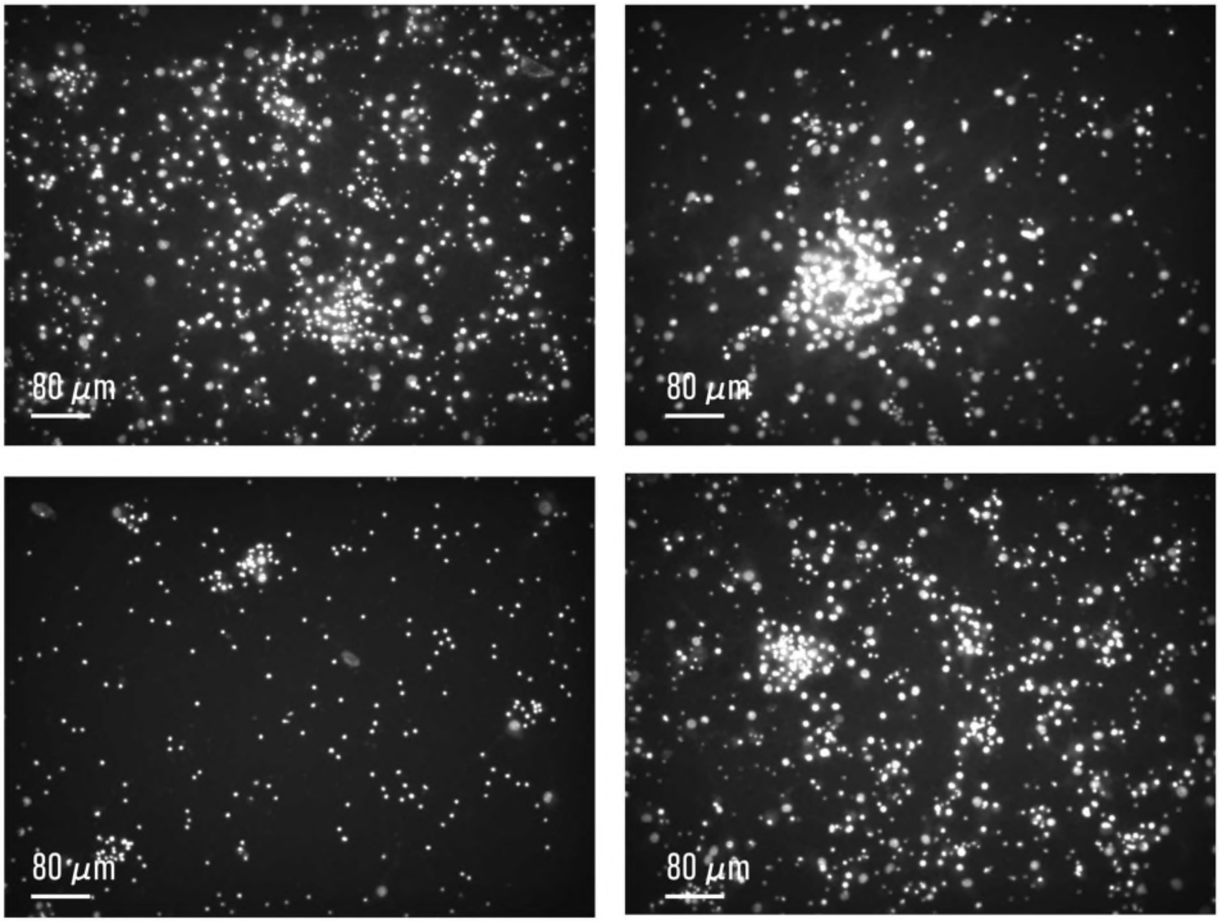

Supplementary Figure 5.3. Selection of fluorescence images of neuronal cells on PDMS surfaces taken $24h$ from culture. In acquiring the images, cell-nuclei were selectively stained with DAPI, here visualized in grayscale colors. ($E = 1\ MPa$).

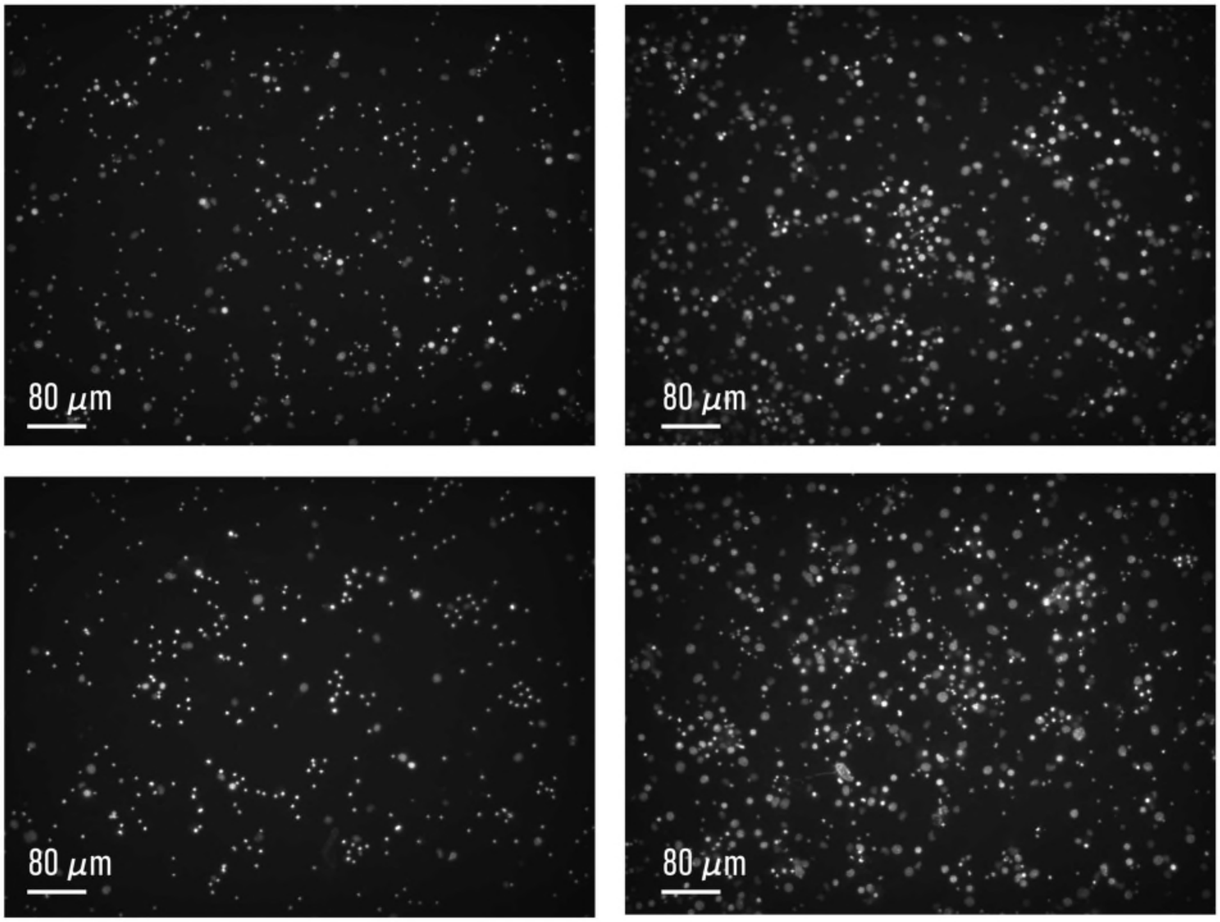

Supplementary Figure 5.4. Selection of fluorescence images of neuronal cells on PDMS surfaces taken $24h$ from culture. In acquiring the images, cell-nuclei were selectively stained with DAPI, here visualized in grayscale colors. ($E = 1.88\ MPa$).

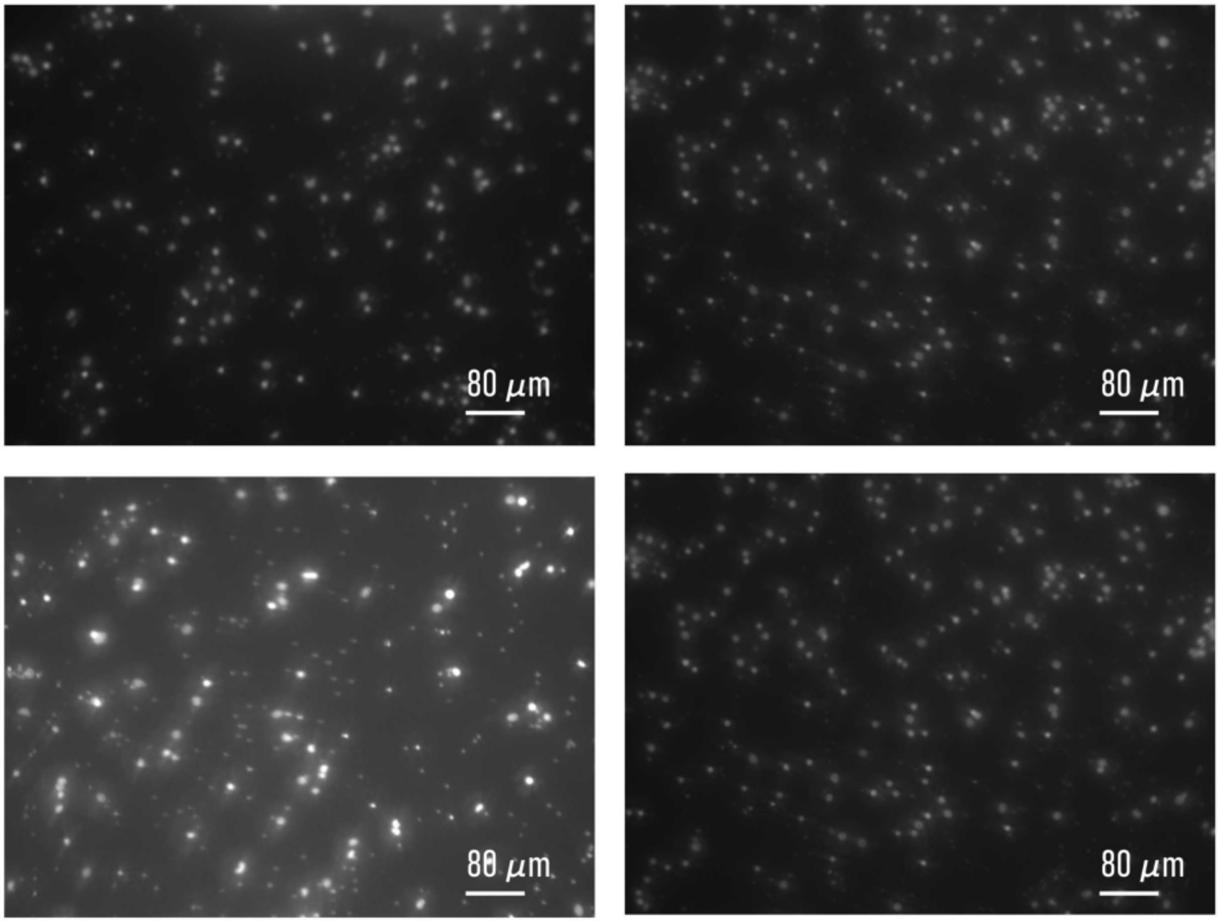

Supplementary Figure 5.5. Selection of fluorescence images of neuronal cells on PDMS surfaces taken $24h$ from culture. In acquiring the images, cell-nuclei were selectively stained with DAPI, here visualized in grayscale colors. ($E = 2.65\ MPa$).

Supplementary Note 6. *Neuronal networks determined from the fluorescence images of cells on soft PDMS surfaces*.

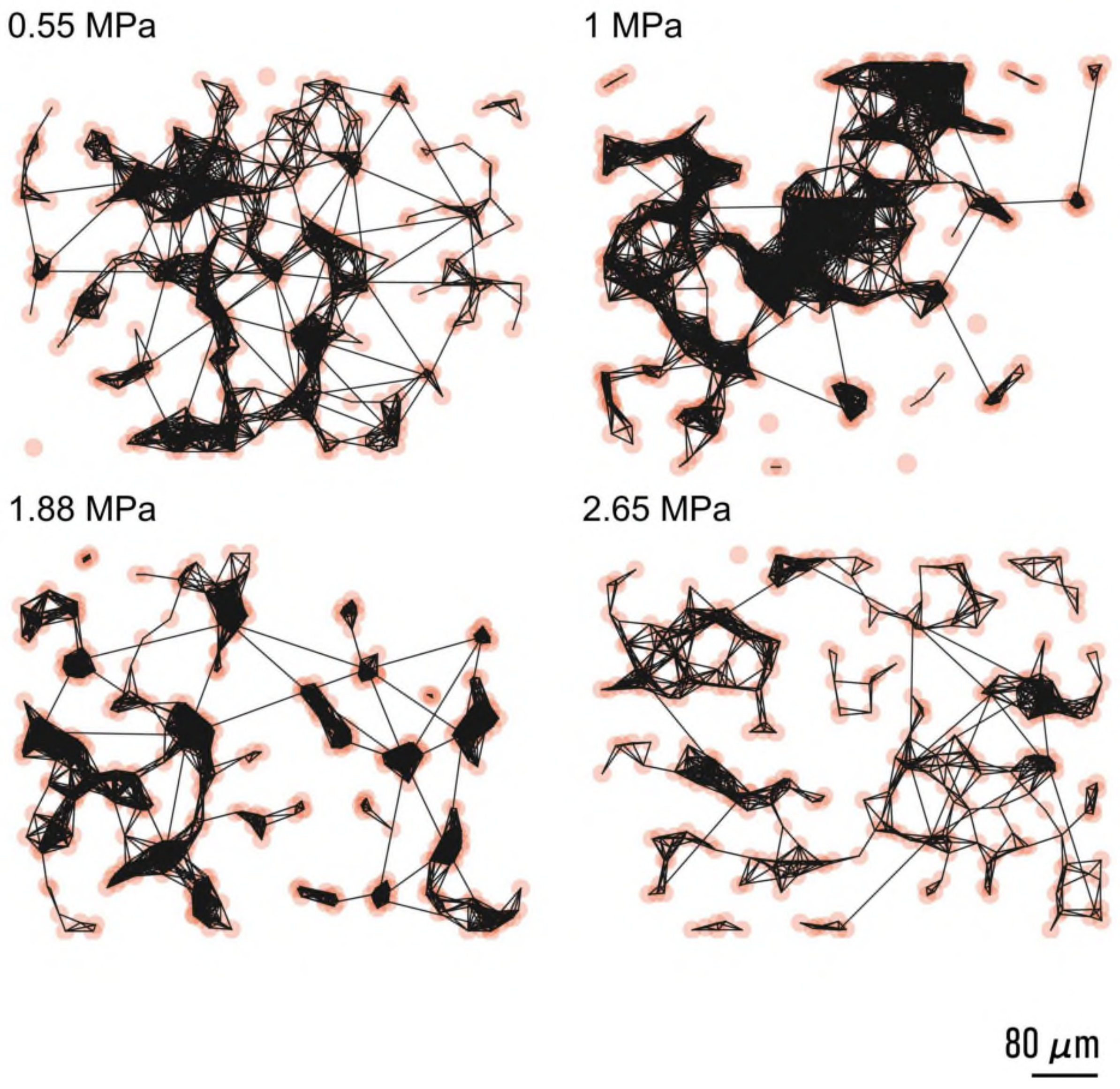

Supplementary Figure 6.1. Networks out of neuronal cells cultured on soft PDMS surfaces imaged $48h$ from culture for different values of the elasticity of the substrate.

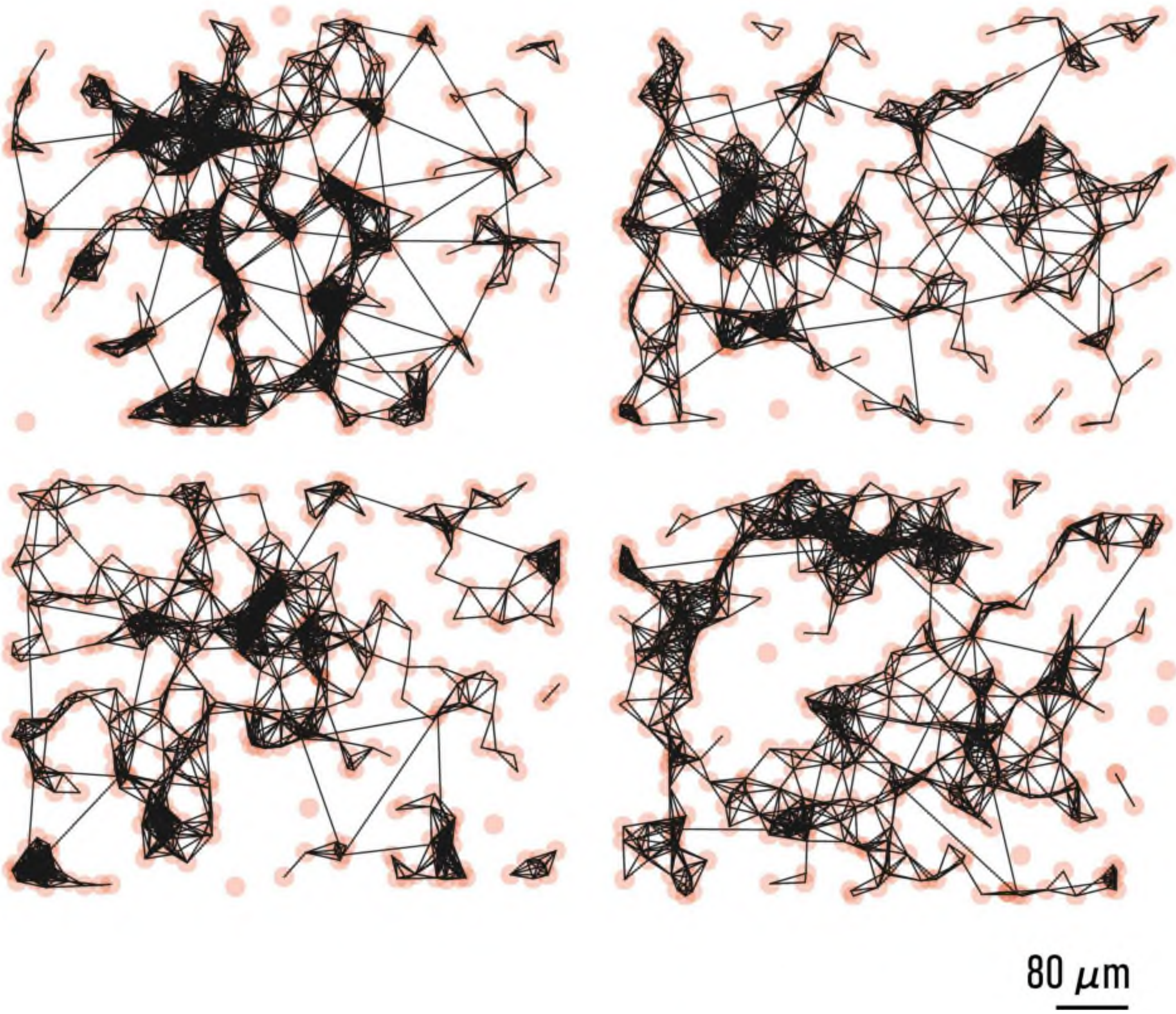

Supplementary Figure 6.2. Networks out of neuronal cells cultured on soft PDMS surfaces imaged $48h$ from culture ($E = 0.55\ MPa$).

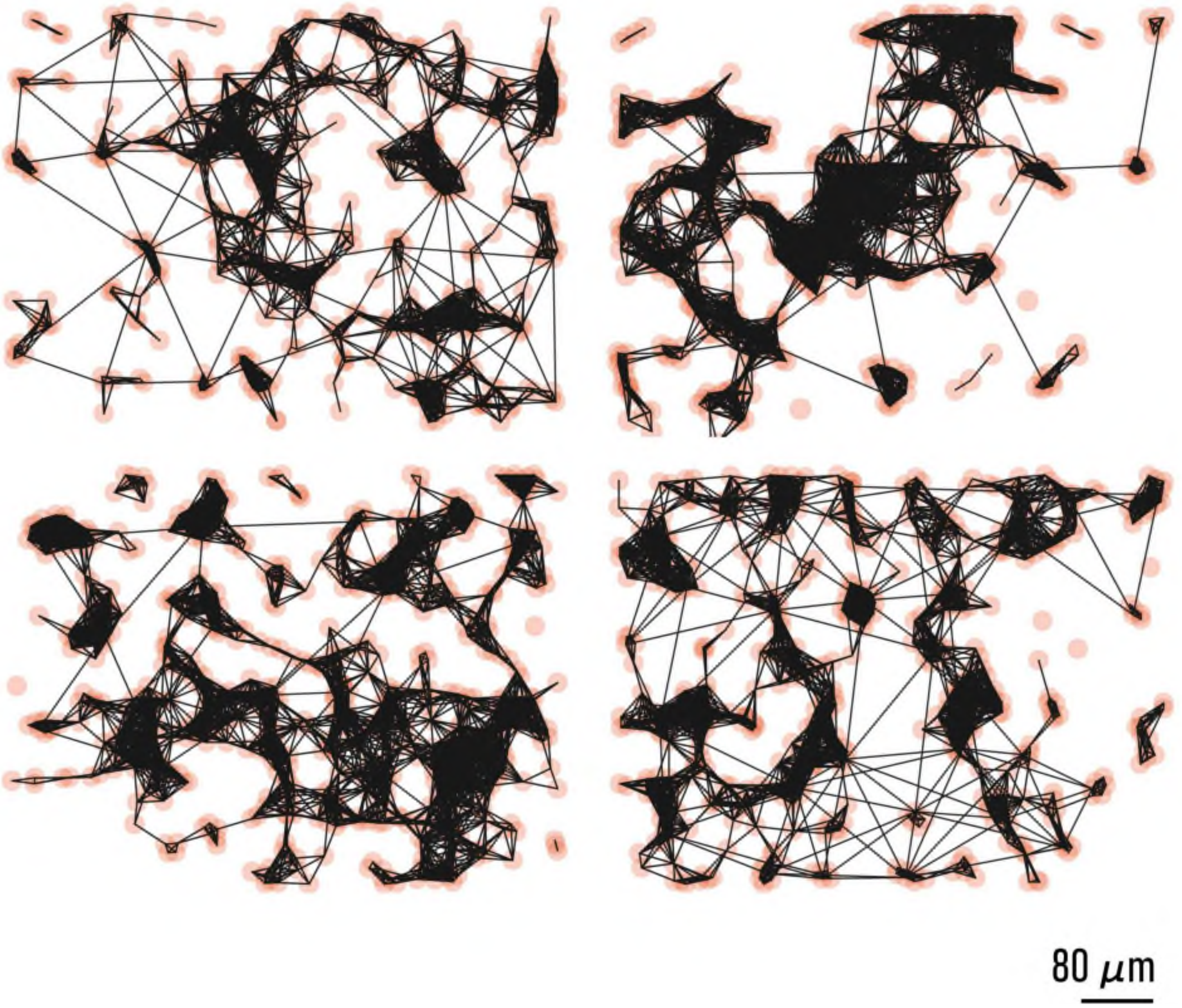

Supplementary Figure 6.3. Networks out of neuronal cells cultured on soft PDMS surfaces imaged $48h$ from culture ($E = 1\ MPa$).

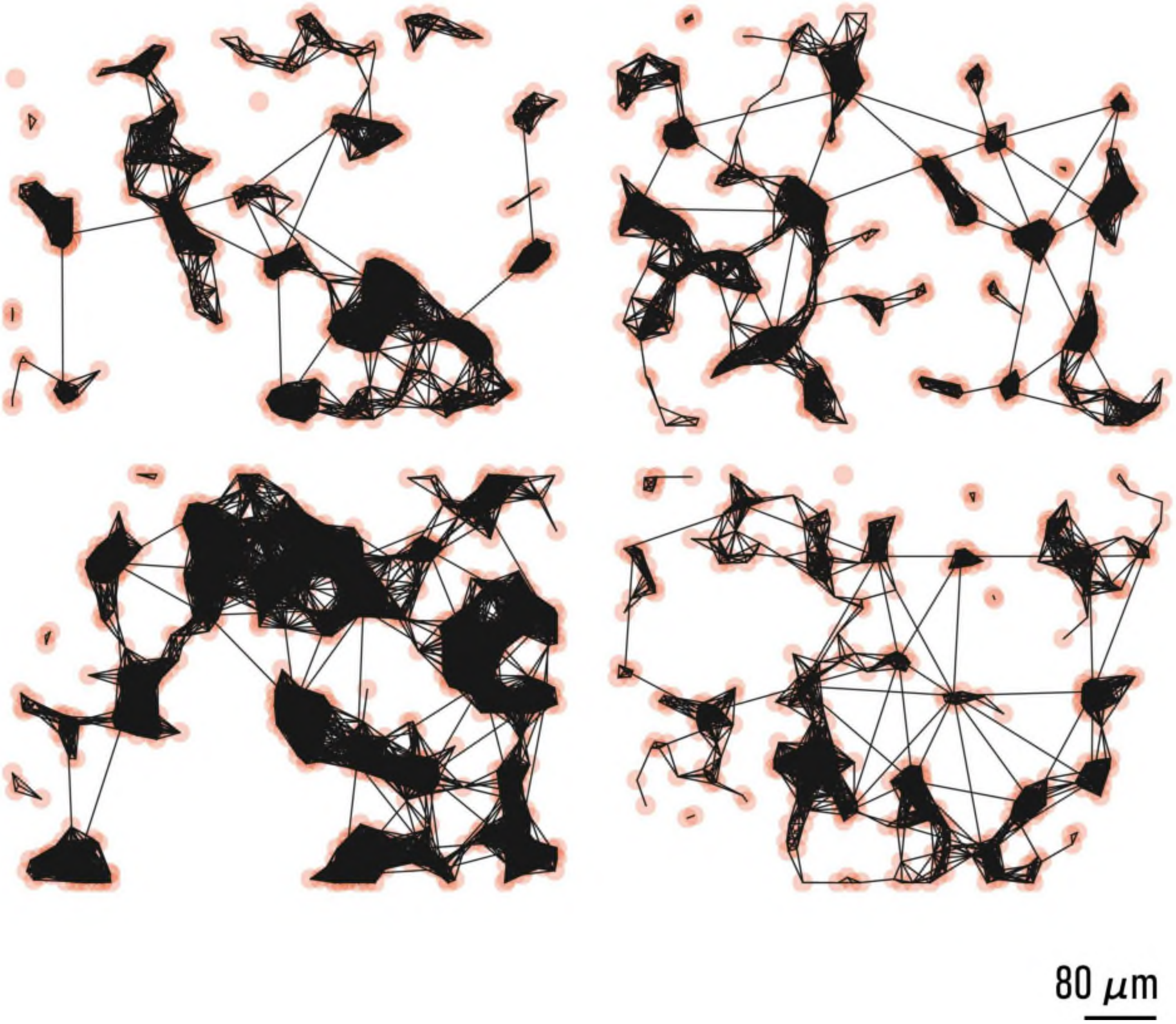

Supplementary Figure 6.4. Networks out of neuronal cells cultured on soft PDMS surfaces imaged $48h$ from culture ($E = 1.88\ MPa$).

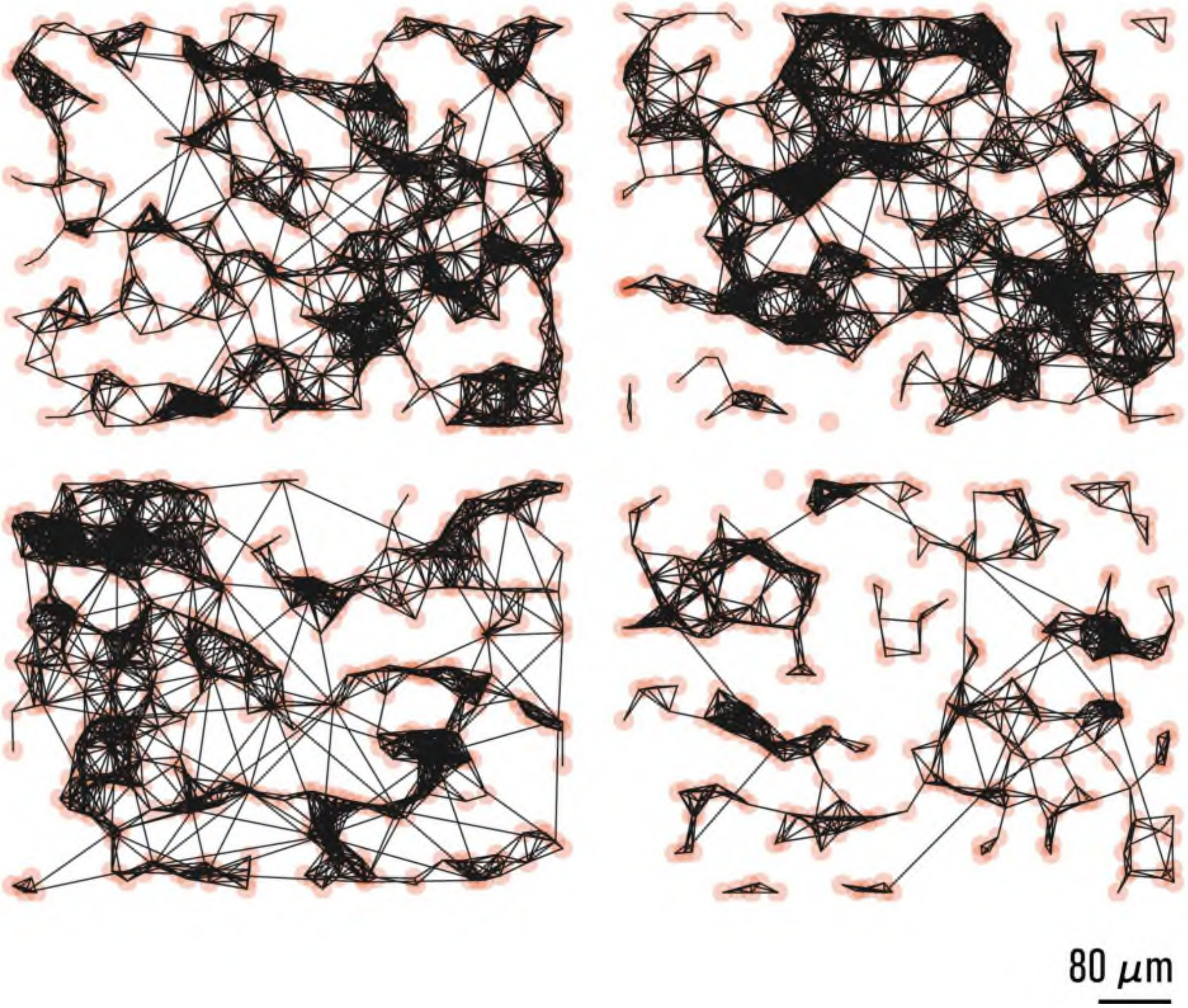

Supplementary Figure 6.5. Networks out of neuronal cells cultured on soft PDMS surfaces imaged $48h$ from culture ($E = 2.65\ MPa$).

Supplementary Note 7. *SW characteristics of neuronal cell graphs for different values of the cut-off probability* p *– a parameter in the cells-wiring model*.

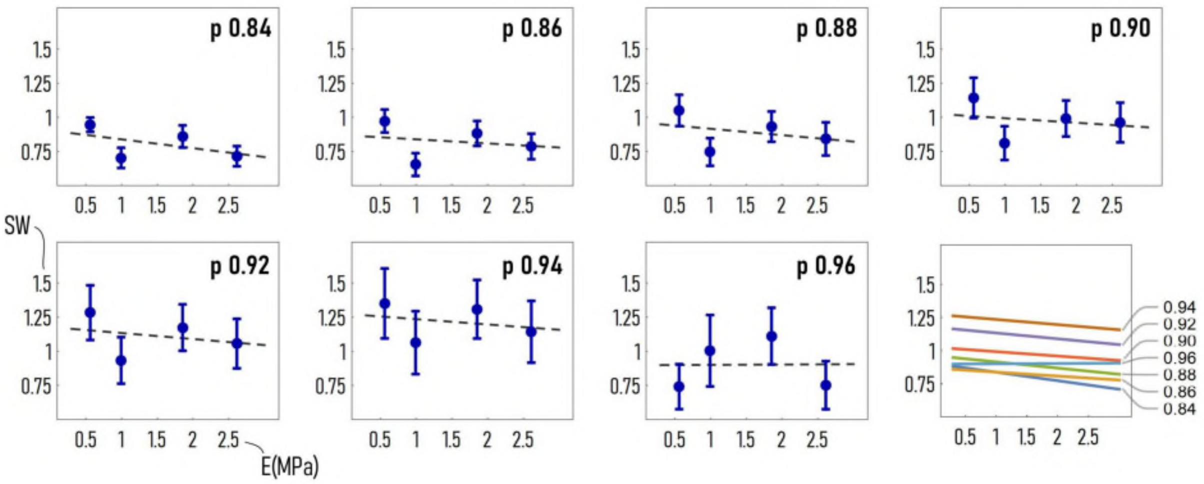

Supplementary Figure 7.1. Small world coefficient of neuronal cell graphs as a function of the elasticity of soft PDMS substrates, for different values of the wiring-model parameter p ($24h$ from culture).

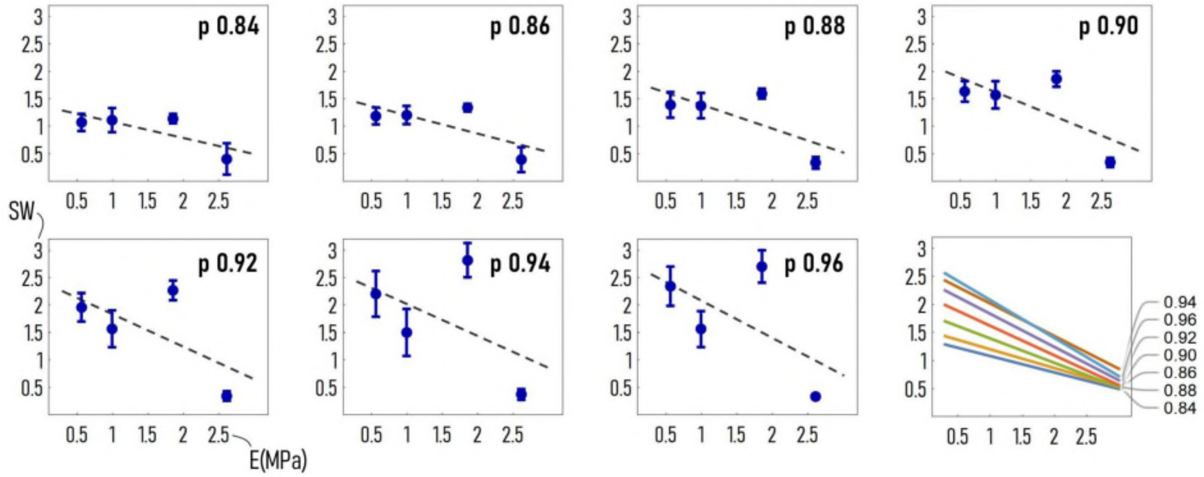

Supplementary Figure 7.2. Small world coefficient of neuronal cell graphs as a function of the elasticity of soft PDMS substrates, for different values of the wiring-model parameter p ($48h$ from culture).

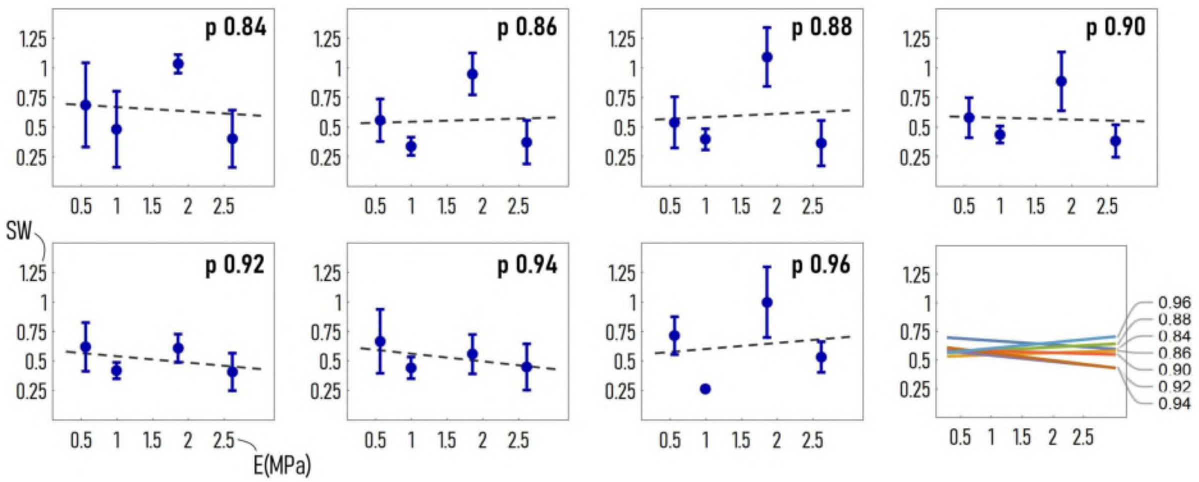

Supplementary Figure 7.3. Small world coefficient of neuronal cell graphs as a function of the elasticity of soft PDMS substrates, for different values of the wiring-model parameter p ($72h$ from culture).

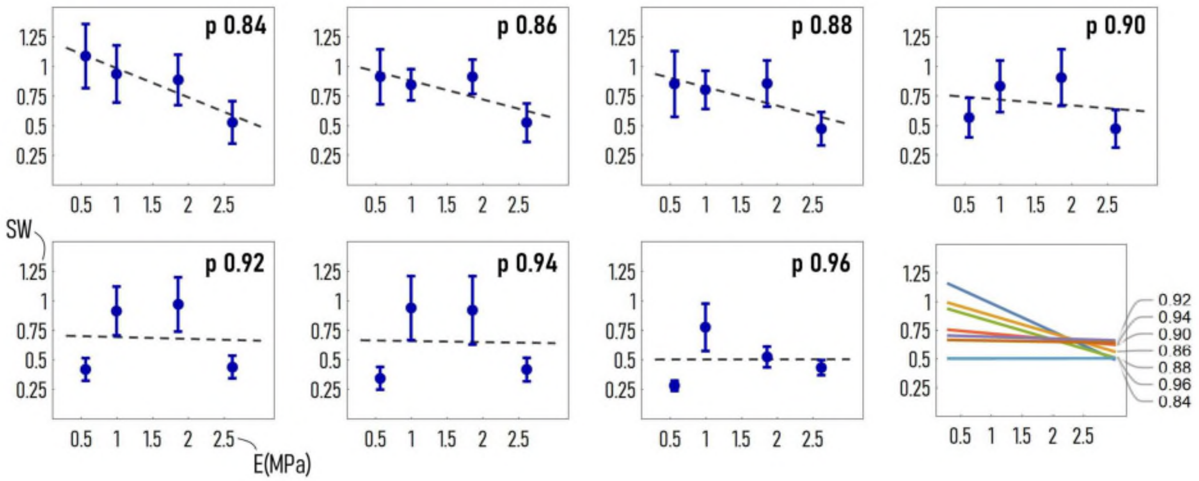

Supplementary Figure 7.4. Small world coefficient of neuronal cell graphs as a function of the elasticity of soft PDMS substrates, for different values of the wiring-model parameter p ($96h$ from culture).

Supplementary Note 8. *Direct reconstruction and characterization of neuronal cell graphs by neuronal branching analysis from green fluorescent images.*

In this study - the topological characteristics of neuronal networks have been determined starting from the positions of the nuclei on the surface - that have been then elaborated through convenient wiring models. However, this represents just an *estimate* of how cells connect. A similar model based on cell-distance may explain less neuronal cell connectivity and more cell-condensation and clustering.

To generate more reliable neuronal cell networks and provide a consolidated reference against which results of the work can be verified, we performed additional analysis. In place of examining cell-nuclei, we analyzed neuronal branching from green fluorescent images of cells, in which actin filaments were labelled using green fluorescent staining phalloidin conjugate – as conveniently reported in the methods of the paper. Since actin filaments are expressed in subcellular structures such as growth cones or dendritic spines, they can be used to dissect neurite outgrowth or synapse physiology.

For different sample characteristics, we examined green-fluorescent images of neuronal cells lining the PDMS surface (**Supplementary Figure 8.1a**). Images were then gray-scale converted and enhanced used a bi-lateral filter (**Supplementary Figure 8.1b**). After correction, images where skeleton-transformed, this enabled to reduce foreground regions in the originating image, preserving the extent and connectivity of the original region while throwing away most of the original foreground pixels (**Supplementary Figure 8.1c**). After removing smaller disconnected objects, the morphological graph of the cells was determined - giving the morphological branch points and endpoints of the image (**Supplementary Figure 8.1d**). The very good overlap of the axons in the originating fluorescent image of cells, with the topological-neural network (**Supplementary Figure 8.1e**) is an evidence of the correctness of the method that we have developed to find the graph-analogue of biological neurons. Then, the topological characteristics of the network g depicted in the **Supplementary Figure 8.1d** and **e** - were compared to those determined for an Erdos-Renyi random graph with the same size of g (**Supplementary Figure 8.1f**) to determine the small-world coefficient of the cells. Thus, this image analysis procedure and algorithm enable to generate a faithful analogue of real neuronal graphs (**Supplementary Figure 8.2**).

For the cells considered in this example (i.e. the control) we found that $SW = 1.004$, practically the same as the value $SW = 1$ estimated in the study through simple neurons-wiring. The same analysis was performed for cells cultured for 48 h on PDMS surfaces with values of elasticity $E = 0.55\ MPa$, $E = 1\ MPa$, $E = 1.88\ MPa$ and $E = 2.65\ MPa$ (**Supplementary Figure 8.3**).

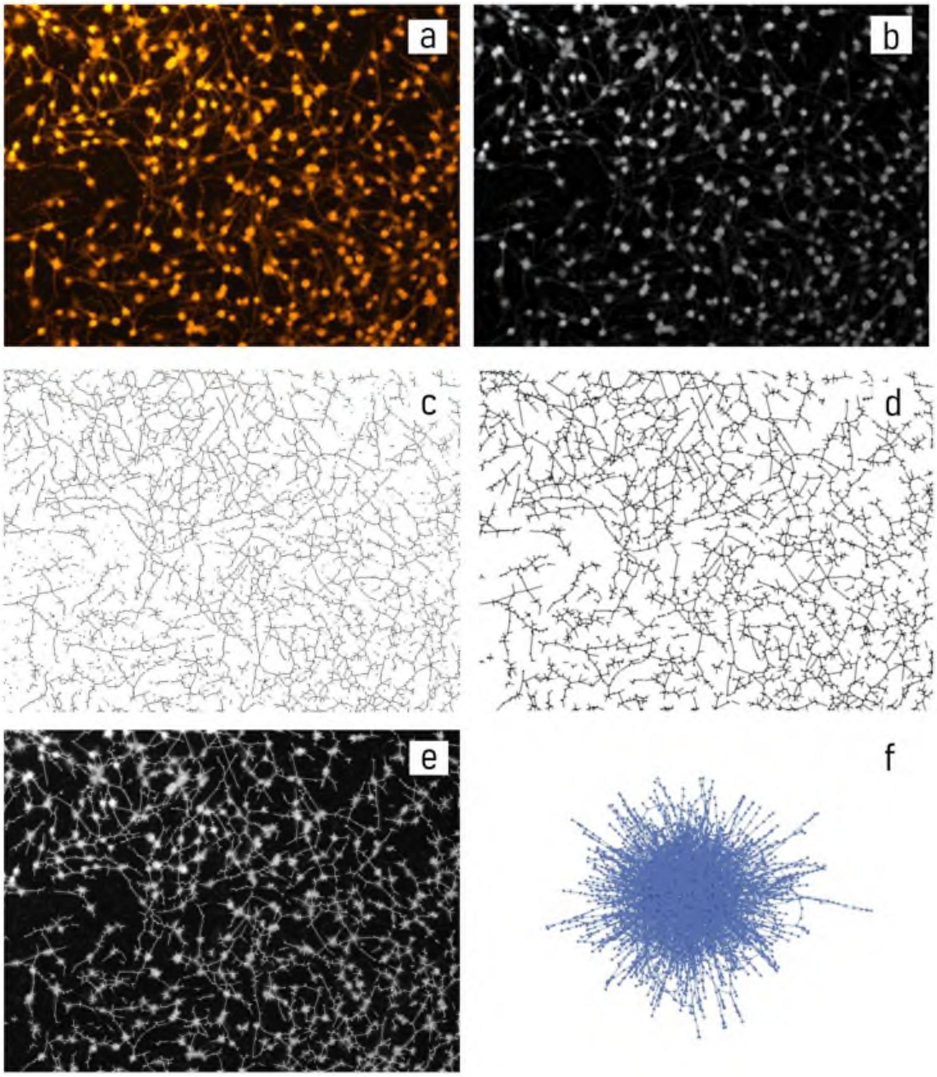

Supplementary Figure 8.1. Image analysis and processing of fluorescent images of cells, aimed at the determining the most faithful representation of neuronal cell networks.

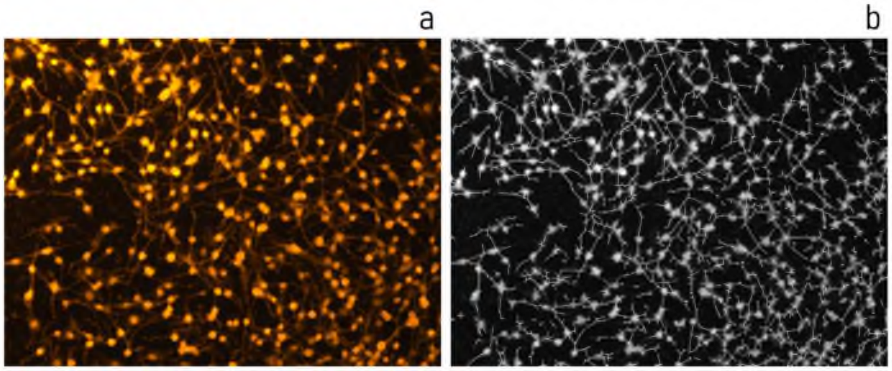

Supplementary Figure 8.2. Neuronal-network analogue (b) of real neurons (a) – determined by the neuronal branching/image analysis algorithm described in the Supplementary Figure 8.1.

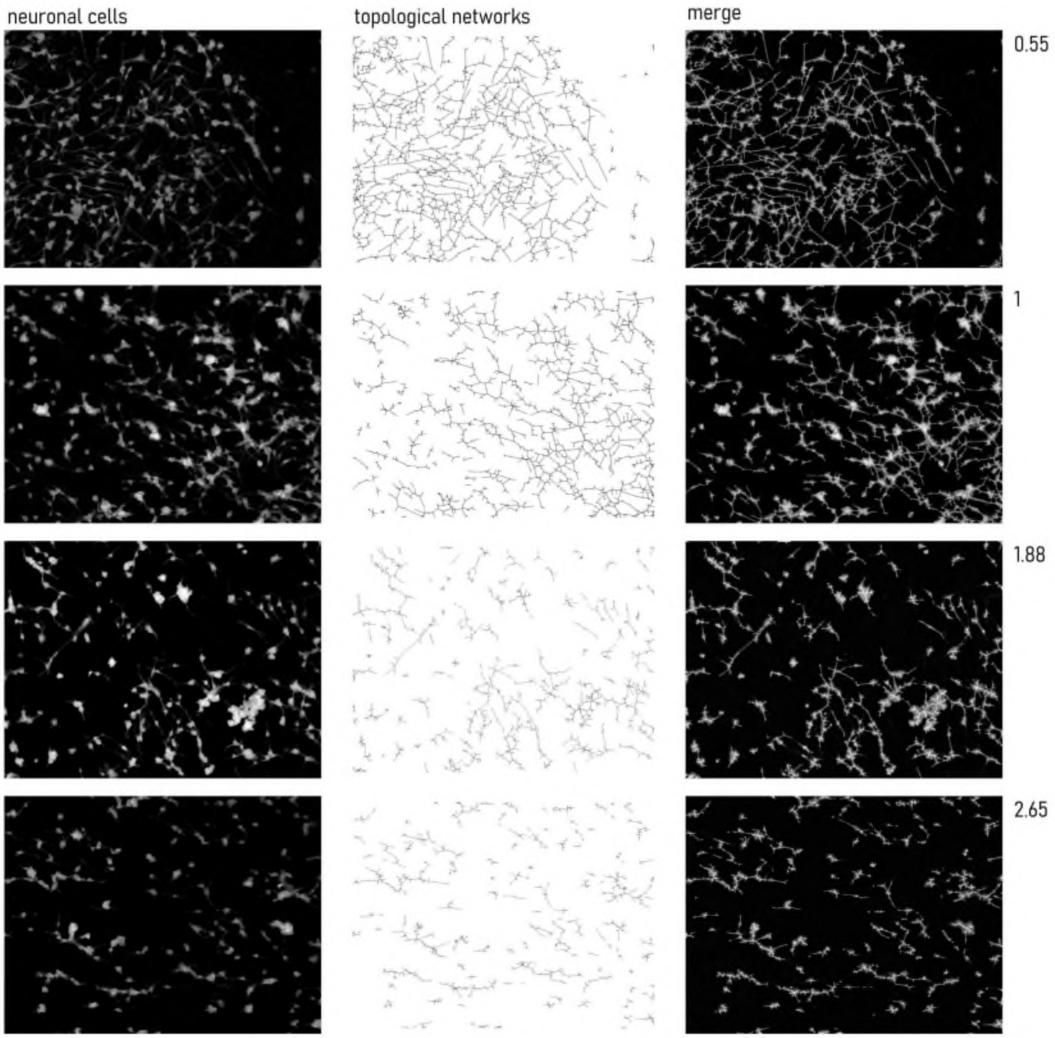

Supplementary Figure 8.3. Image analysis and processing performed on cells sitting on surfaces with different values of elasticity and polymeric-base:curing-agent mixing ratio.

Results illustrate that the small-world coefficient of neuronal cell graphs decreases linearly with the Young's modulus (**Supplementary Figure 8.4**) – in line with the results already presented in the study obtained by connecting nodes using a mixed distance and density rule wiring model of cells.

Specifically, the linear law that correlates the small-world coefficient (sw) to the Young's modulus of PDMS surfaces (E) obtained using the Waxman model on neurons - imaged 48h from culture - is $\mathrm{SW} = 2.42 - 0.59\,E$. In contrast, the relationship found by more-accurate image analysis and processing of fluorescent images of neuronal cells – is $\mathrm{SW} = 1.98 - 0.62\,E$.

The very close resemblance between linear model fits obtained using different procedures indicates that results of this research based on a cell-cell distance and cell-density wiring model of cells – are accurate.

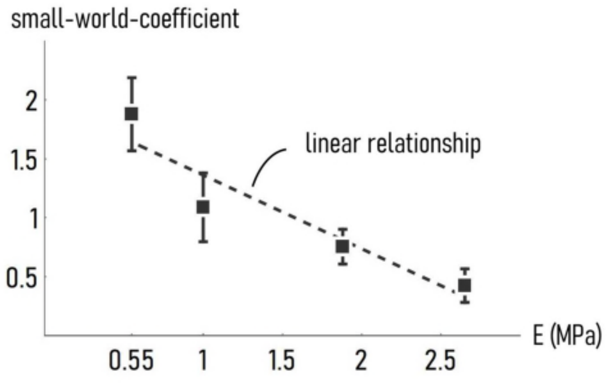

Supplementary Figure 8.4. Linear relationship between the small world-coefficient of neuronal networks determined from fluorescent images of cells, and the elasticity of PDMS cell-culture substrates.

Supplementary Note 9. *Characterization of PDMS leakage into DI water.*

We have performed an additional test campaign aimed at characterizing leakage of PDMS into DI water at different times and under different temperatures. To do so, we used both Raman spectroscopy and energy dispersive X-ray spectroscopy (EDX).

As regarding Raman analysis.

PDMS substrates, molded into cylindrical shapes with a diameter of 2 cm and a height of 0.5 cm using varying ratios of the liquid PDMS to curing agent ratio (r: 1, r = 7, 10, 13, 15, 18), were submerged in 200 µl of water to assess their stability. The testing was conducted at two different temperatures: 37°C, to mimic cell culture conditions, and 60°C, to accelerate the leaching process. Additionally, the substrates were incubated for a time varying from 24 h (short incubation period) to 108 h (long incubation period). To analyze any products that may have been released following the long-standing exposition of PDMS to water, a drop from each solution was placed in duplicate on a CaF2 slide and on a CaF2 slide coated with a sputtered gold layer, then allowed to dry (**Supplementary Figure 9.1**). The samples were subsequently examined using a Renishaw inVia Raman microscope (Renishaw, Turin, Italy) at ambient temperature, employing a 50 × objective of a Leica microscope (Leica Micro Systems, Wetzlar, Germany). Raman spectra were collected by exciting the samples with a 630.0 nm laser line, at a laser power of 2.5 mW and an integration time of 10 to 20 s.

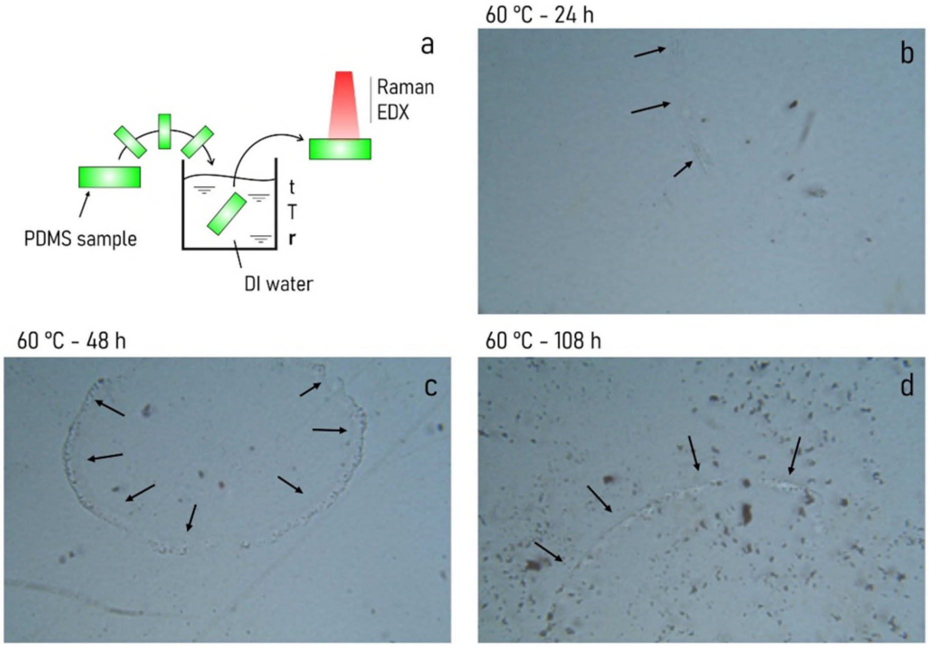

Supplementary Figure 9.1. *Experimental scheme.* PDMS discs were exposed to DI water for a time varying from 24 h to 108 h, setting two different values of the leaching temperature, i.e. $T_1 = 37\ °C$ and $T_2 = 60\ °C$, and for different values of the liquid base:curing agent ratio r. The solution resulting from the

prolonged contact was collected, deposited on a substrate and left evaporate. The residue was then examined by Raman and EDX spectroscopy (*a*). Optical images of PDMS traces released into DI water, for different values of the PDMS-water exposure time and for a fixed leaching process temperature $T = 60\ °C$ (*b-d*).

Analysis of PDMS residue using simple CaF2 substrates fails to detect clear signatures of sample leakage, probably due to the low amplification effects of CaF2, the weak signal associated to PDMS byproducts, and to unwanted fluorescence interference (**Supplementary Figure 9.2-9.3**). Raman spectra reported in the **Supplementary Figure 9.2** are relative to samples obtained under a fixed temperature of T=37°C after 48 and 108 h of exposition. Raman spectra reported in the **Supplementary Figure 9.3** are relative to samples obtained under a fixed temperature of $T = 60°C$ after 48 and 108 h of exposition. In both cases, Raman spectra exhibit no evident peaks attributable to PDMS components in the remnant of solution after exposure, sample collection, and evaporation.

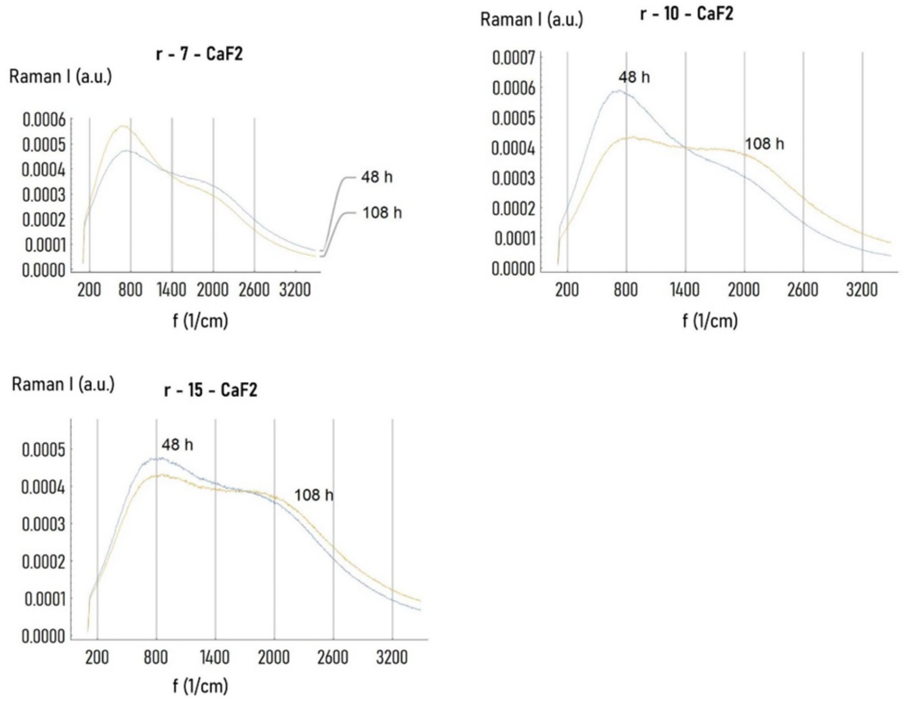

Supplementary Figure 9.2. Raman signature of PDMS traces, for different values of the leaching time, and of the liquid base:curing agent ratio r. For a fixed value of the leaching temperature $T_1 = 37°\ C$.

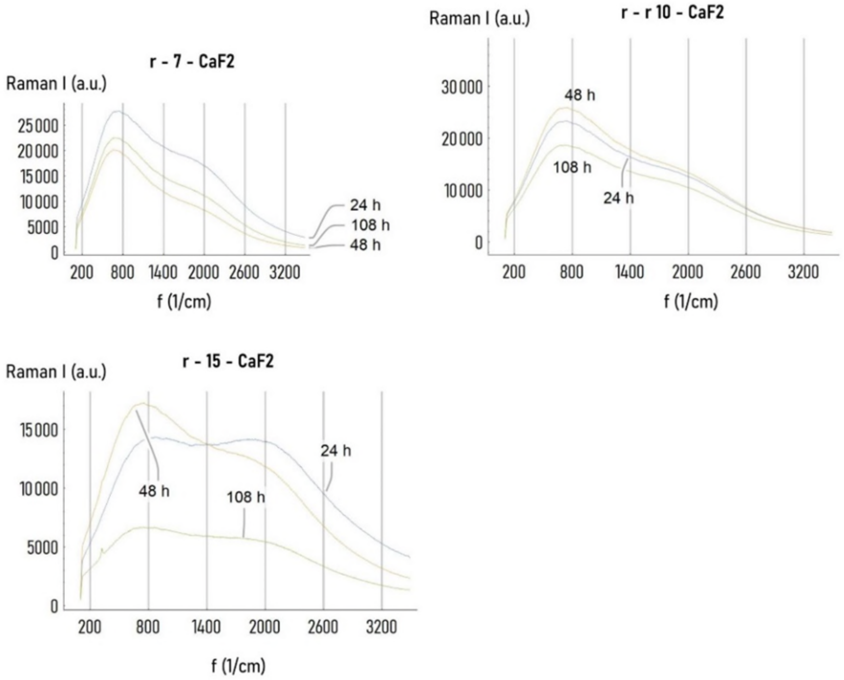

Supplementary Figure 9.3. Raman signature of PDMS traces, for different values of the leaching time, and of the liquid base:curing agent ratio r. For a fixed value of the leaching temperature $T_2 = 60°$ C.

To amplify even more the signal and generate significant Raman spectra, we used SERS (Surface Enhanced Raman Spectroscopy) substrates, obtained by sputtering a discontinuous layer of gold nanoparticles upon an originating CaF2 flat surface (**Supplementary Figure 9.4**).

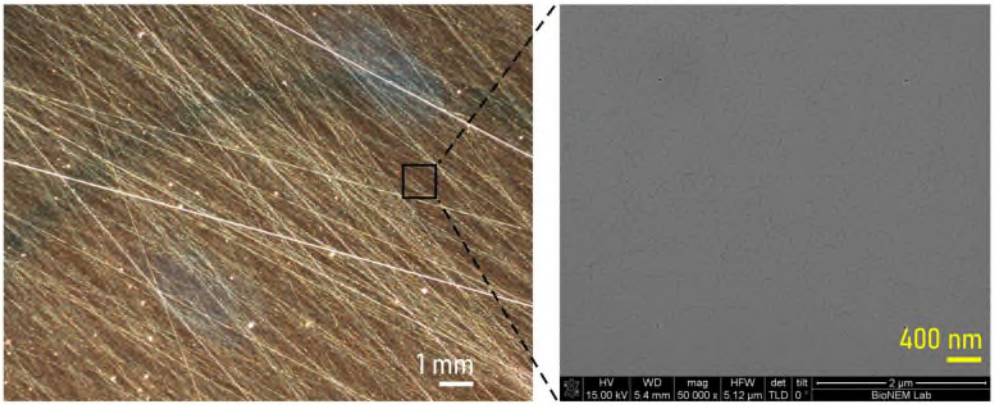

Supplementary Figure 9.4. Optical and SEM image of a CaF2 substrate patterned with gold nanoparticles.

Raman spectra of samples measured on similar SERS surfaces deliver more information about the PDMS components released over time and under different working temperatures, compared to conventional CaF2 substrates. Results of SERS measurements are reported in the **Supplementary**

Figure 9.5 for leaching times of 48 and 108 h, for a PDMS-base/curing agent ratio (r) of $r = 7$, 10, 13, and 18; and for a fixed external temperature $T = 37°C$. After acquisition, Raman spectra were background corrected and normalized to the maximum value in the measurement range. Results of the analysis illustrate that there are peaks characteristic of PDMS traces in solution, specifically:

- Peaks at 784 1/cm and 830 1/cm. While weak, they show an increasing trend from the r=7 to r=18 samples. This trend can be attributed to the Si-C stretch, as indicated in reference (*15*).
- Peak at 1051 1/cm: This peak exhibits a decreasing trend from the $r = 7$ to $r = 18$ samples. It is likely attributed to the C-C stretch, as documented in reference (*15*), possibly due to the methyl residue from the curing agent.

In the **Supplementary Figure 9.5**, for the central frequency 784 1/cm (Si-C stretch) we report the mean-peak intensity as a function of the PDMS to curing agent ratio, r: a quantitative measure of how the Raman signature of PDMS samples varies as a function of sample preparation, for both the 48h and 108h time steps. After 48h of leakage, peak variations across different r's are moderate, with the intensity varying of about the 8% for r moving from $r = 7$ to $r = 13$, and of about 4% for r moving from $r = 13$ to $r = 18$. After 108h of leakage, the variation of intensity relative to the 784 1/cm frequency remains moderate, with the signal varying of about the 5% for r moving from $r = 7$ to $r = 18$.

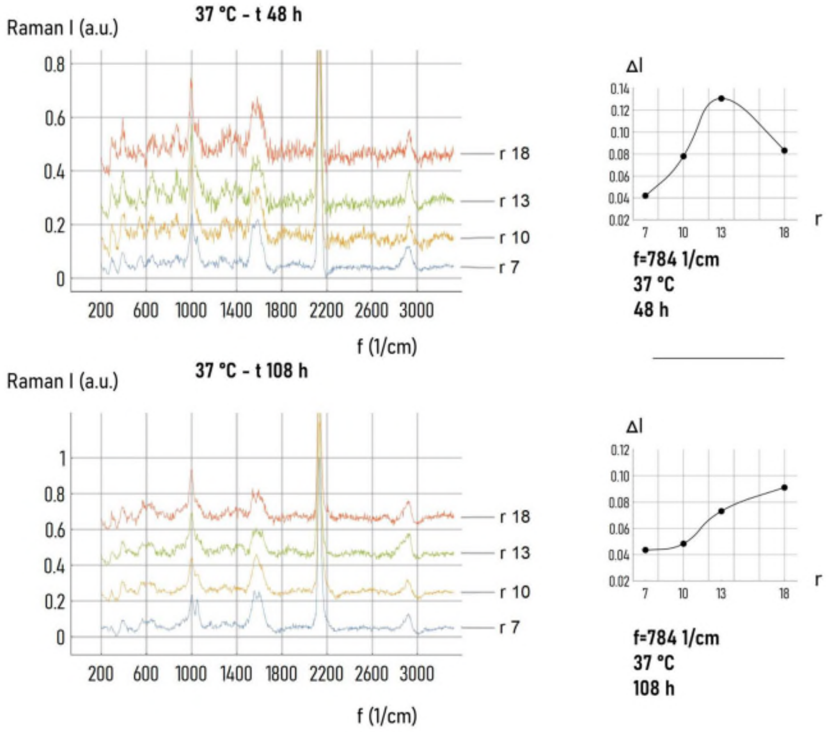

Supplementary Figure 9.5. Raman signature of PDMS traces, for different values of r and of the leaching time (48 h, 108 h), for a fixed leaching temperature $T_1 = 37$ °C. In the figure, we also report the percentage

variation of the Raman peak (ΔI) relative to the frequency $f = 784\ cm^{-1}$, determined as a function of r for both of the considered times of leaching, $48\ h$ and $108\ h$.

Supplementary Figure 9.6 illustrates the same analysis reported in the **Supplementary Figure 9.5**, except that the temperature of the leakage process is set to T=60 °C. For this configuration, the Raman signal measured at 784 1/cm (typical of Si-C stretch) varies of less than the 3% in the 7-18 PDMS:curing agent interval for a 48 h test time, and of a vanishingly small 1% for a 48 h test time.

Results of the PDMS leakage tests performed by Raman analysis suggest that sample preparation (i.e. the ratio r) affects more relevantly the mechanical characteristics of samples (i.e. the Young's modulus), while it influences only moderately the leakage of PDMS in a liquid solution.

This analysis enhances our confidence that the results of the work and the observed, peculiar behavior of neuronal cells are directly related to the mechanical properties of PDMS samples, rather than being influenced by other factors such as leakage.

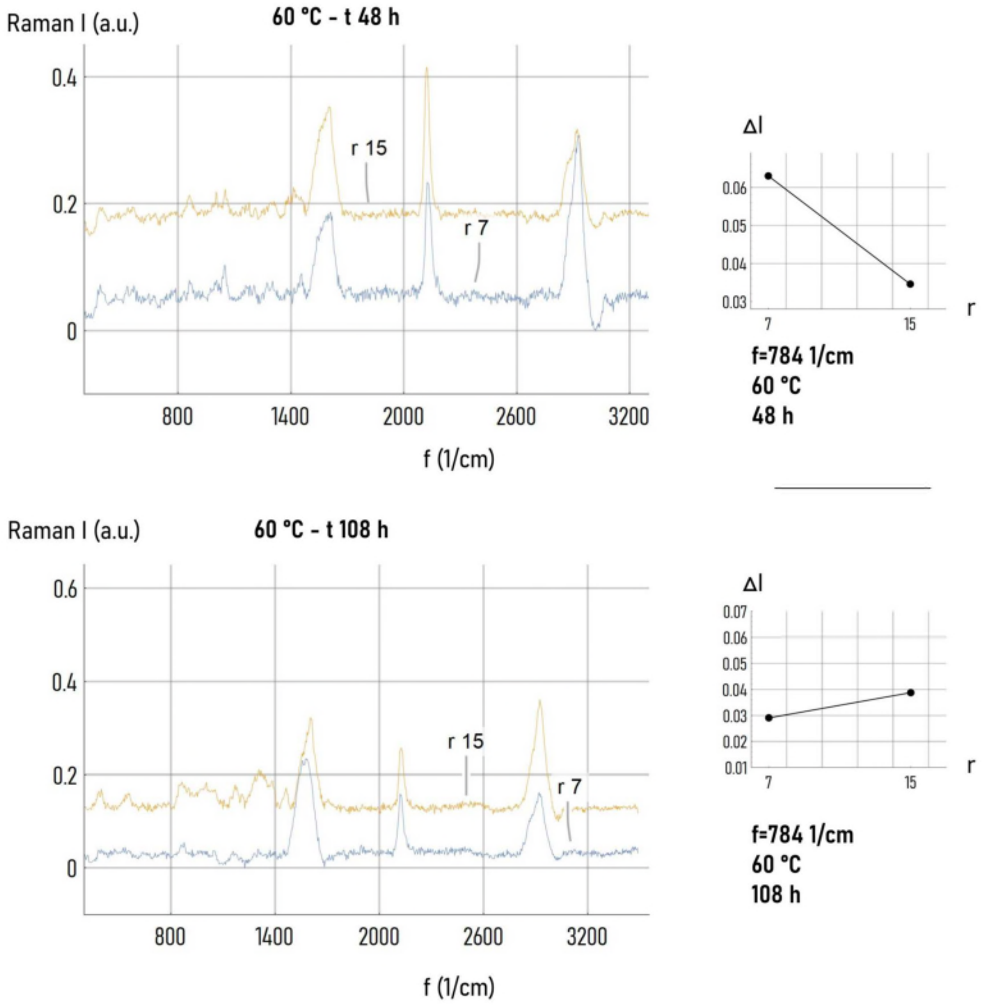

Supplementary Figure 9.6. Raman signature of PDMS traces, for different values of r and of the leaching time ($48\ h$, $108\ h$), for a fixed leaching temperature $T_2 = 60$ °C. In the figure, we also report the percentage variation of the Raman peak (ΔI) relative to the frequency $f = 784\ cm^{-1}$, determined as a function of r for both of the considered times of leaching, $48\ h$ and $108\ h$.

Since the Raman analysis that we have performed is sensitive more to the molecular composition of samples and less to its elements, we carried out an additional sample test by using energy dispersive X-ray spectroscopy (EDX). Differently from Raman spectroscopy, this technique is sensitive to the elements constituting the samples. Moreover, EDX can be used to estimate the relative abundance of elements in a sample. To perform the tests, a 20 µl drop of DI-water in contact with PDMS under different conditions was deposited on a clean standard SEM pin stub and analyzed by FESEM ULTRA-PLUS (Zeiss) (Milan, Italy) with the SE2 detector.

We performed the analysis on samples exposed at different leakage temperatures (37 °C, 60 °C), for different incubation times (24 h, 48 h, 108 h), and with different initial PDMS:curing agent ratio (r: 7, 10, 13, 15). Results of the analysis are reported in the **Supplementary Figures 9.7** to **9.13**.

Supplementary Figure 9.7 and **9.8** illustrate results of an EDX analysis performed on samples obtained from the interaction between DI water and PDMS, following 48 h (**9.7**) and 108 h (**9.8**) of exposure at a steady incubation temperature of T=37°C, in relation to the liquid base-to-curing agent ratio, r.

Similarly, **Supplementary Figure 9.9** to **9.11** illustrate results of an EDX analysis performed on samples obtained from the interaction between DI water and PDMS, following 24 (**9.9**), 48 (**9.10**) and 108 h (**9.11**) of exposure at a steady incubation temperature of T=60°C, as a function of the liquid base-to-curing agent ratio, r.

Results illustrate that the main elements found in the solution at contact with PDMS for prolonged amount of times are Carbon, Oxygen, and Silicon, that is consistent with preliminary analysis performed by Raman analysis and with independent reports (*16, 17*). In particular, Oxygen is, by far, the most abundant element in the PDMS residue, followed by Carbon and Silicon. At 37 °C (a temperature of interest since it is similar to that used in the cell-experiments reported in the work) none of the considered elements correlate with r. As for an example, for a leakage time of 48 h, the relative abundance of Si varies from ~9% for $r = 7$, to ~4% $r = 10$, to ~16% for $r = 13$. For a leaching time of 108 h, the content of silicon in the residual solute is of ~5% for $r = 7$, of ~2.5% for $r = 10$, of ~2.7% for $r = 13$.

Considering a higher value of temperature T=60 °C, we observe that the abundance of Si decreases with r for 24 h and 48 h leaching times, while it oscillates with r between the 1.7 % and 2.2 % values for a 108 h leaching time.

The dependence of the relative of abundance of Si on time and temperature is illustrated in the **Supplementary Figure 9.12** and **9.13**.

Supplementary Figure 9.12 shows that, for a fixed temperature T=37°C, the relative amount of Si in the final solution decreases linearly with time, with a slope that is relevant for r=15, less relevant and for r=7, and negligible for r=10 (again: the rate of change of [Si] with T, does not correlate with r). Conversely, for a fixed temperature T=60°C, the relative amount of Si in the final solution oscillates with time, and reaches a maximum at 48 h. Notably, the relative abundance of Si at 48 h is higher for r=7, followed by r=10, and by r=15. Thus, at 37°C and 60°C r seems to play different roles, enhancing (hampering) the release of Si at higher (lower) temperature.

Also the temperature (T) seems to have opposite effects on the content of Si, depending on the leaching time (**Supplementary Figure 9.13**). At 48 h, [Si] increases with T. At 108 h, [Si] decreases with T. For both 48 and 108 h, the rate of change of [Si] with T is more relevant for $r = 7$, and less relevant for $r = 10$.

Thus, results of Raman and EDX analysis, collectively, indicate that the leakage of PDMS may be less relevant than the mechanical properties of substrates in determining cell behavior, for reasons that can be summarized as follows:

1. The Raman signal of PDMS traces is, in any case, vanishingly small, and could be detected only through SERS effects. This indicates that leakage of PDMS is negligible.
2. The variation of Raman signal associated to Si-C stretching is small for varying values of r, that in turn indicates that the liquid-base:curing-agent ratio influences only moderately leakage.
3. EDX analysis of samples illustrates that, in a given amount of PDMS excess, the relative abundance of Silicon correlates poorly with r – similarly to other elements found in solution. This indicates that, when present, the effects of leakage cannot explain the enhanced adhesion and enhanced clustering of neurons – that is instead related to the inverse of r.
4. Even assuming a significant release of Si into water or the culture medium used for neurons, silicon, in the form of silicon dioxide, or silicon-based nano- and micro-particles, and particulate, is generally considered to be biocompatible and not toxic to cells under many conditions (*18*, *19*). The biocompatibility of Si-based nano- and micro-scale materials has been a focus of ongoing research efforts to understand the factors affecting their interactions with biological systems.

Considering all this, we confidently rule out that leakage is responsible of the peculiar cell behavior observed and reported in our work, either alone or combined with other mechanisms or PDMS characteristics, such as elasticity.

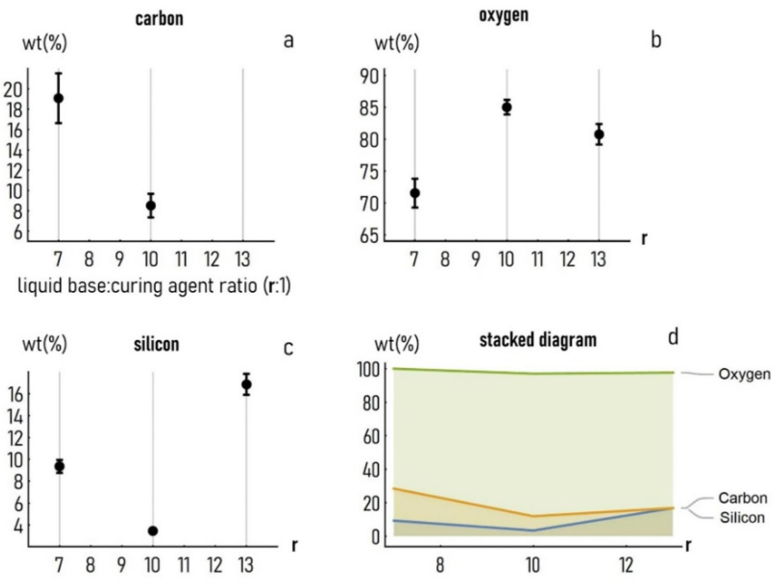

Supplementary Figure 9.7. EDX analysis of samples resulting from the interaction of DI water and PDMS, after an exposition of $\sim 48h$ at a constant incubation temperature of $T_1 = 37°C$, as a function of the liquid base:curing agent ratio r.

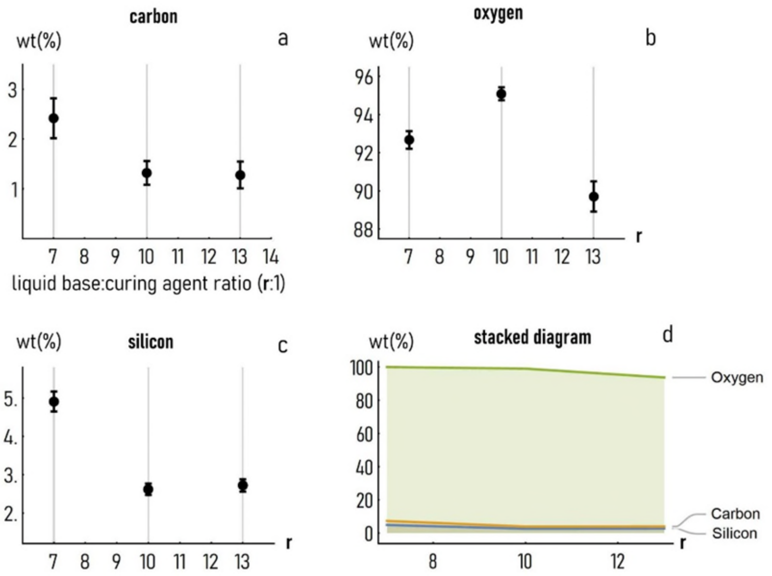

Supplementary Figure 9.8. EDX analysis of samples resulting from the interaction of DI water and PDMS, after an exposition of $\sim 108h$ at a constant incubation temperature of $T_1 = 37°C$, as a function of the liquid base:curing agent ratio r.

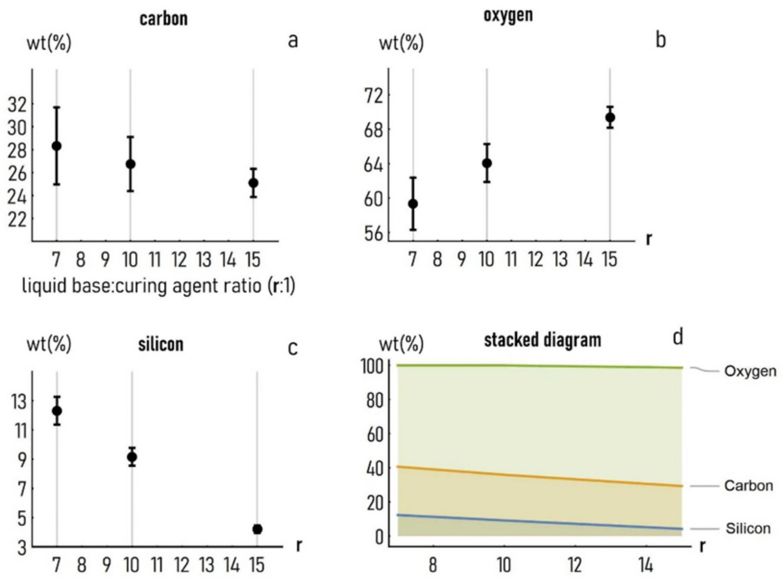

Supplementary Figure 9.9. EDX analysis of samples resulting from the interaction of DI water and PDMS, after an exposition of $\sim 24h$ at a constant incubation temperature of $T_2 = 60°C$, as a function of the liquid base:curing agent ratio r.

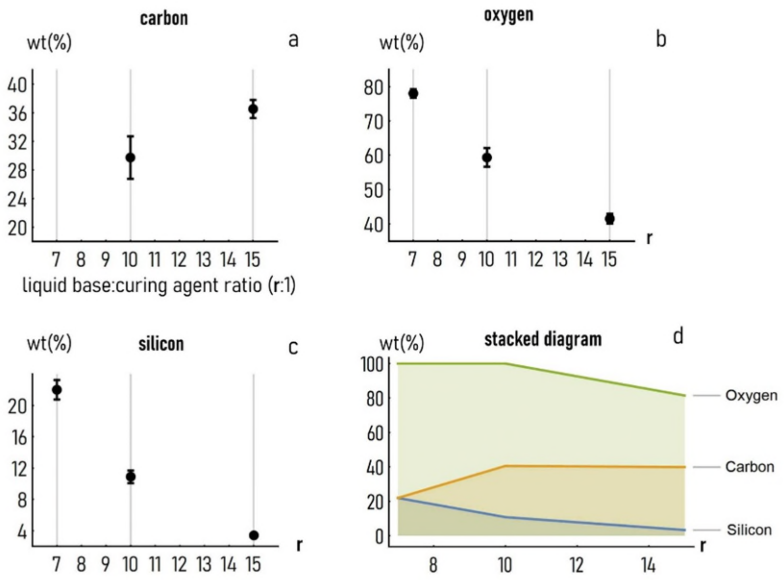

Supplementary Figure 9.10. EDX analysis of samples resulting from the interaction of DI water and PDMS, after an exposition of $\sim 48h$ at a constant incubation temperature of $T_2 = 60°C$, as a function of the liquid base:curing agent ratio r.

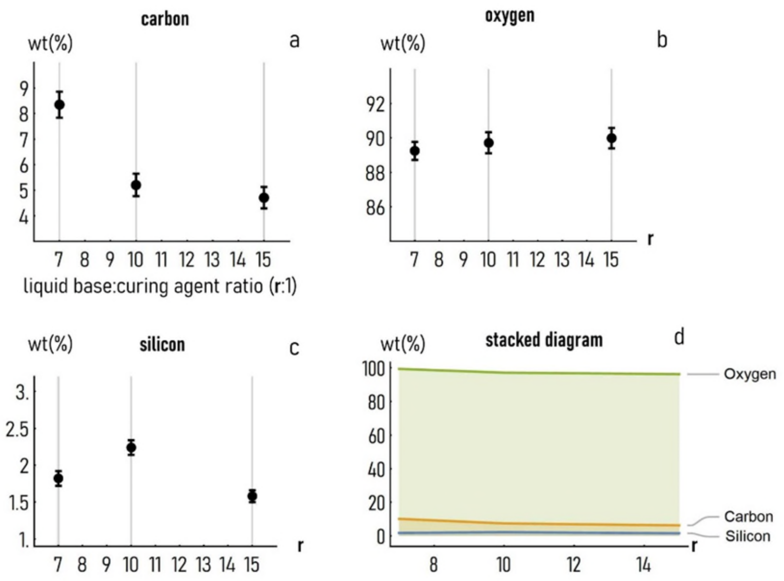

Supplementary Figure 9.11. EDX analysis of samples resulting from the interaction of DI water and PDMS, after an exposition of $\sim 108h$ at a constant incubation temperature of $T_2 = 60°C$, as a function of the liquid base:curing agent ratio r.

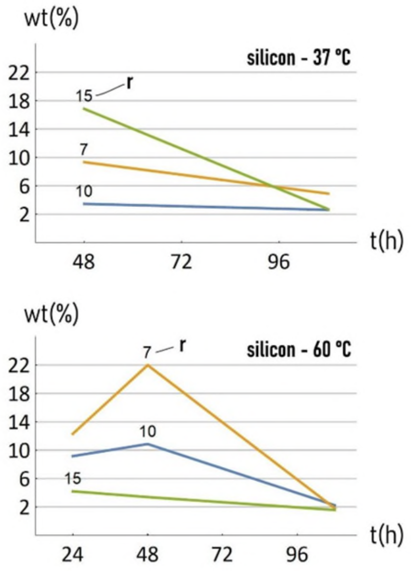

Supplementary Figure 9.12. Relative content of silicon in the solution resulting from the exposure of DI water with PDMS, ad a function of *time*, for different values of the liquid base:curing agent ratio r.

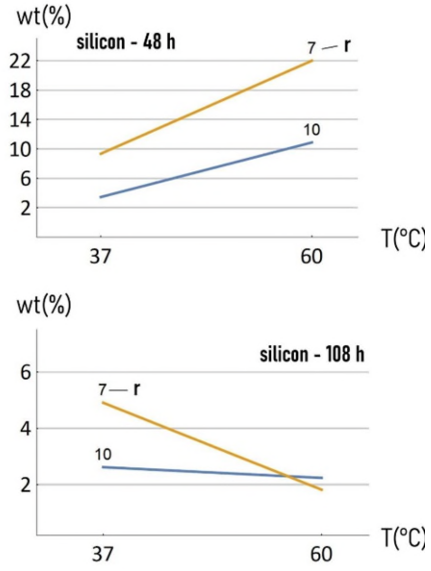

Supplementary Figure 9.13. Relative content of silicon in the solution resulting from the exposure of DI water with PDMS, ad a function of *temperature*, for different values of the liquid base:curing agent ratio r.

Supplementary Note 10. *A mathematical model of adhesion and clustering on soft surfaces*.

The mathematical model that we have developed is an attempt to explain the observed increased adhesion and clustering of cells on soft PDMS surfaces. Consider the scheme in the **Supplementary Figure 10.1**. Cells adhere to a rough soft surface.

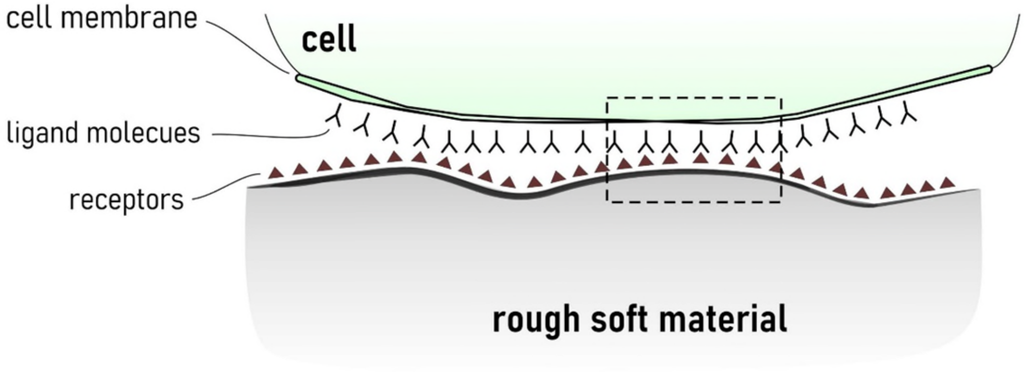

Supplementary Figure 10.1 *Cartoon depicting the adhesion of a cell on a soft nanorough surface.*

Adhesion is described by the energy term γ: the surface energy density of adhesion that accounts for both specific (i.e. mediated by cell adhesion molecules) and non-specific (steric, van Der Waals, electrostatic) interactions (*20*, *21*) – the larger γ, the firmer the adhesion of a cell on the surface. Following adhesion, cell generates forces that act on the surface. The intensity of such forces can be estimated after the Johnson-Kendall-Roberts (JKR) model (*22*, *23*) as

$$F_{jkr} = \frac{3}{2}\pi R w, \qquad (10.1)$$

where $R(x)$ is the radius of curvature of the surface and $w = \gamma$ is the work of adhesion. Thus, F_{jkr} is a local force and its intensity depends on the *morphology* of the substrate measured at the position x. Consider, for sake of illustration, a random rough surface $y(x)$ with average roughness 20 nm, i.e. the same value of roughness measured for the PDMS surfaces used in this study (**Supplementary Figure 10.2**). For this profile, the radius of curvature $R(x)$ is calculated after the equation:

$$R(x) = \left(1 + \left(\frac{dy}{dx}\right)^2\right)^{3/2} \Bigg/ \left|\frac{d^2y}{dx^2}\right|, \tag{10.2}$$

and is reported in the **Supplementary Figure 10.3**.

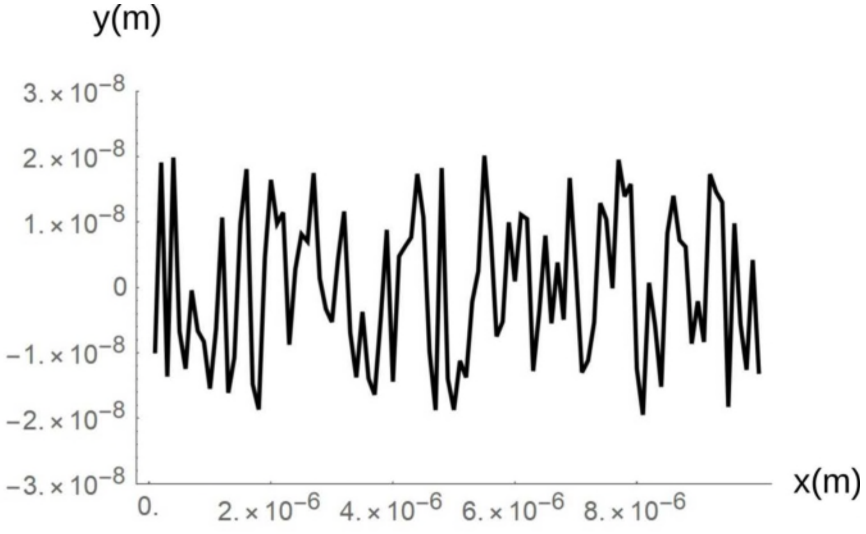

Supplementary Figure 10.2 *Random rough profile describing the morphology of soft substrates.*

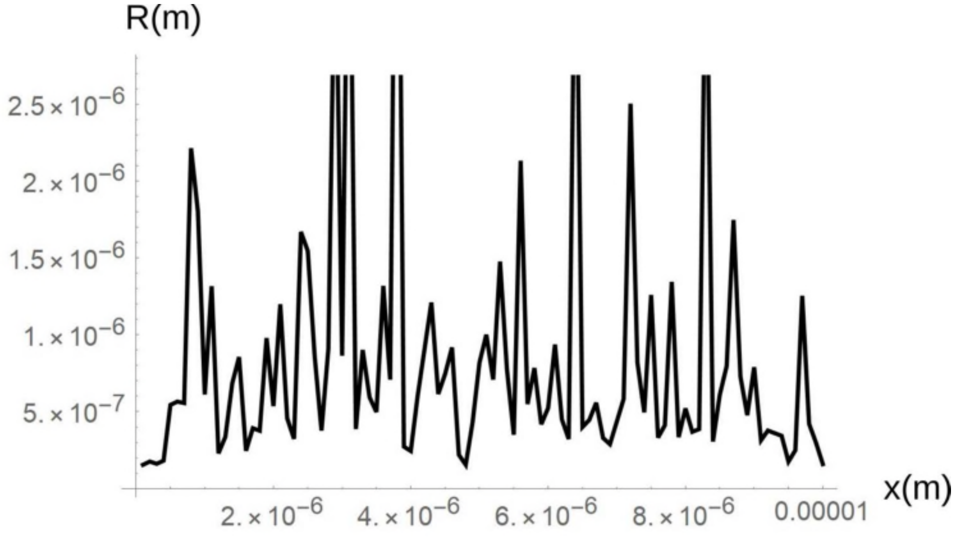

Supplementary Figure 10.3 *Radius of curvature of the random rough profile reported in the Supplementary Figure 10.2.*

One can notice that $R(x)$ is non-uniform - the intrinsic irregularities of $y(x)$ are amplified. As a result, the forces pushing on the surface calculated through equation (10.1) are also irregular (**Supplementary Figure 10.4**). For this configuration and for $\gamma = 10^{-5}\ J/m^2$, values of the F_{jkr} force of the cell on the surface range from 0 to $\sim 0.2\ nN$. Since the substrate is soft, it deforms under external loads. Points of the surface are then displaced in the direction of the F_{jkr} force of the extent:

$$\delta y(x) = y(x)\ \frac{F_{jkr}(x)}{E\ A}, \tag{10.3}$$

where E is the Young's modulus of the soft PDMS surface and A is the cross sectional area of the elements into which the original profile is discretized (**Supplementary Figure 10.4**). The morphology of the substrate following deformation is then described by the function $y_{fin} = y_{in}(1 - \delta y/y)$.

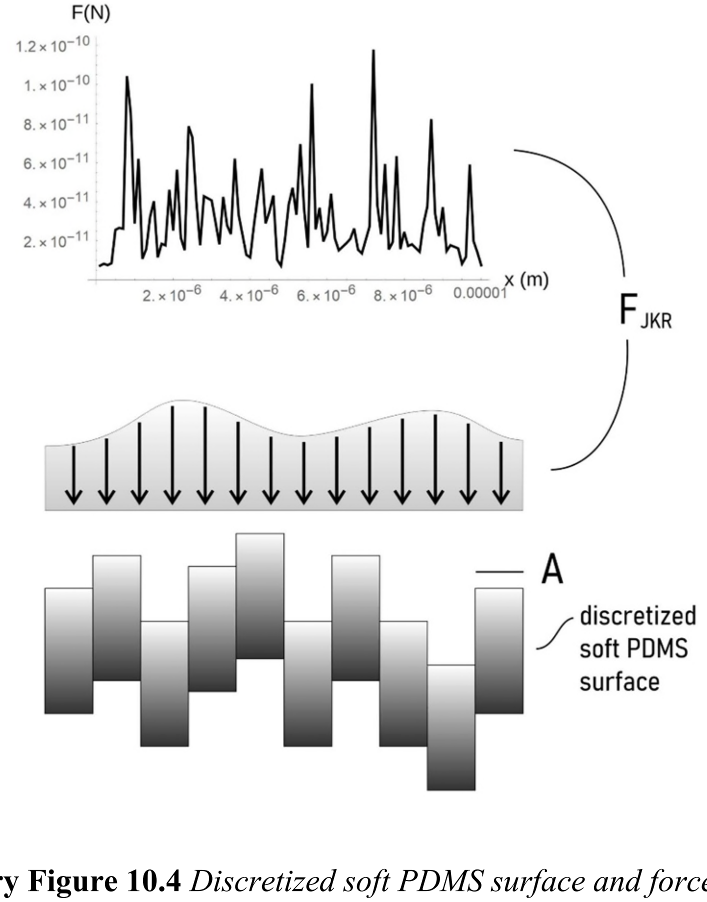

Supplementary Figure 10.4 *Discretized soft PDMS surface and forces acting on it.*

For the values of γ, E and A used in this simulation, we found that the morphology of the surface after adhesion (y_{fin}) is significantly different from the initial shape (y_{in}) (**Supplementary Figure 10.5**; $\gamma = 10^{-5}\ J/m^2$, $E = 1$ MPa, $A = 10\ nm \times 10\ nm$). Notably, the value of surface roughness *increases* from Ra$\sim 20\ nm$ (y_{in}) to Ra$\sim 30\ nm$ (y_{fin}).

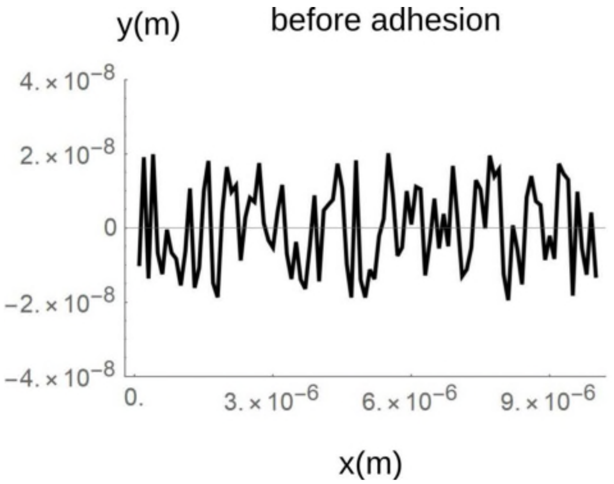

Supplementary Figure 10.5 *Comparison of the morphologies of the soft PDMS substrate before (left) and after (right) cell adhesion.*

However, if roughness increases - also the surface area increases. Under the hypothesis of firm adhesion, this implies that the surface area available for *adhesion* increases. This, in turn, has as a consequence an enhancement of the *effective* specific energy of adhesion γ_{eff} between the cell and the surface (*20*). The enhancement of γ_{eff} (with respect to the initial profile with γ_o) can be estimated with Decuzzi and Ferrari as (*20*):

$$\frac{\gamma_{eff}}{\gamma_o} = -\frac{2}{\gamma} E_{cell}\, \lambda\, U, \qquad (10.4)$$

where

$$U = \frac{\gamma}{2\pi}(E_{cell}\,\lambda)^{-1}\,G(2\pi a, -h^2\pi^2) + \frac{\pi^2}{12}\left(\pi^2 a + \frac{\pi}{4}sin(4\pi a) + \frac{sin^2(2\pi a)}{1-2a}\right)h^2 s^3 + \frac{1}{4\pi^2}(G(2\pi a, -h^2\pi^2) - 2\pi a)^2 s. \tag{10.5}$$

In equations (10.4-10.5): E_{cell} is the Young's modulus of the cell membrane, λ is the wavelength of the substrate (i.e., the characteristic grain size measured on the surface), G is the elliptic integral function of the second type, s is the non-dimensional membrane thickness, and a is the portion of substrate in close contact with the cell membrane. Moreover, h is a measure of the increased roughness of the substrate $h = (\mathrm{Ra}_2 - \mathrm{Ra}_1)/\lambda$.

For $E_{cell} = 1\,kPa$, $\lambda = 20\,nm$, $s = 0.1\,\lambda$, and $\gamma = 10^{-5}\,J/m^2$, the diagram of γ_{eff}/γ_o as a function of h is reported in the **Supplementary Figure 10.6**. For these values of the model parameters, to an increment of roughness of 50% is associated an enhancement of the strength of adhesion of about 45%.

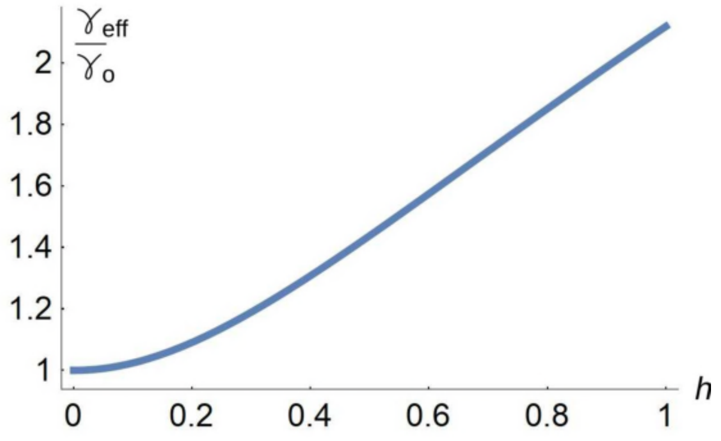

Supplementary Figure 10.6 *The diagram describes how the effective specific energy of adhesion increases as a function of the non-dimensional increment of roughness.*

Thus, the system evolves into a configuration described by an equivalent specific energy of adhesion that is about 1.45 times the value before adhesion: $\gamma_2 {\sim} 1.45\,\gamma_1$ and, assuming $\gamma_1 =$

$10^{-5}\ J/m^2$, then γ_2~$1.45\ \times 10^{-5}\ J/m^2$. An increased energy of adhesion has effects also on the lateral forces that emerge at the cell-surface. With Gentile (*24*), these forces can be estimated as:

$$F_x = -\frac{\partial U}{\partial a}, \tag{10.6}$$

and, since U depends on γ, any change of the specific energy of adhesion may influence F_x.

For the values of the variables that we have used in the simulations, using equation 10.6 we found that the intensity of the lateral force on cells varies from 1 to about 2 nN, upon deformation of the substrate and relative enhancement of roughness. A two-fold increase of the force that pulls on cells can, with Gentile (*24*), mark the transition from non-clustered to clustered systems.

Supplementary Note 11. *Colocalization analysis between neurons and the overall cell population on conventional flat surfaces.*

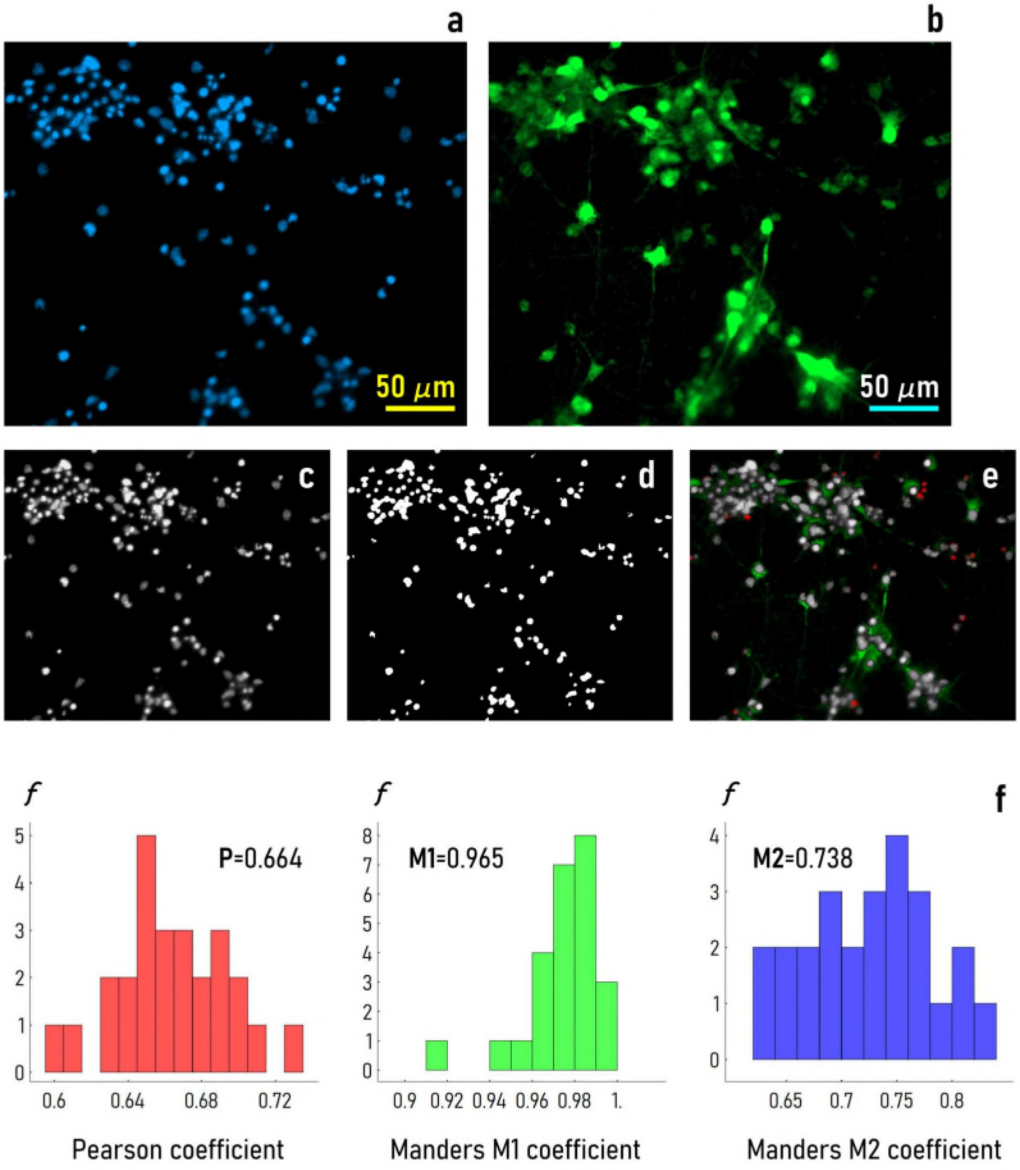

Supplementary Figure 11.1. Colocalization between the signal of fluorescence relative to mouse hippocampal cells (*a*) and hippocampal neurons (*b*). Before the colocalization process, cells were grayscale converted (*c*). Then, in each image the foreground and the background were separated by setting an automatic threshold (*d*). Following colocalization, the spatial overlap between the fluorescent labels associated to the cell and the neuron, was determined (*e*). Colocalization results are described in terms of quantitative parameters, such as the Pearson correlation coefficient, the Manders M1 and M2 coefficients (*f*).

Supplementary Note 12. *Determining the fractal dimension of PDMS surfaces*.

The topography images of soft PDMS surfaces were Fourier transformed and circular averaged around the centers of the images. Results of the computation were reported in a log-log plot as a function of space frequency, and represent the power spectrum (PS) density function of the originating topography images. The PS describes how the morphological characteristics of the surface change per change of scale. The PS exhibits a linear behaviour in defined frequency ranges. The derivative β of the PS in these ranges may be correlated to fractal dimension of the surfaces as as $D_f = (8 - \beta)/2$.

Supplementary Note 13. *Cell image analysis and processing.*

Fluorescent images of cells were imported in Matlab R2020b and converted from RBG to gray scale format. The k-means algorithm was applied to segment the images and select the sole region occupied by cells. The k-means clustering algorithm partitions the originating images into k different segments (*25*, *26*). The information content of the image was associated to one of the segments and all the other segments were disregarded as background. The segment containing the information was shifted to black pixels (binary 1), while the background was associated to white pixels (binary 0). k depends upon the particular problem at study and, for the present configuration, it was set as $k = 8$. After segmentation, a grid with a mesh size of 8×8 pixels was applied to the images to determine the average intensity color of each mesh and associate this value to the probability of being a cell. A threshold (80% of the maximum color intensity) was applied to define the presence of a cell. The region was shrunk to a single pixel and associated with a node, corresponding to the center of the cell.

Supplementary Note 14. *Neuronal cells wiring.*

Once that the neuronal cell centers were determined, they were connected using a mixed distance and density rule described elsewhere (*27*, *28*). The algorithm makes a decision on whether cells on a substrate are connected on the basis of their distance and local density. If points of the dataset (i) are closer to each other than a constant or (ii) they have relative high density and long distances from points with higher density, then they are linked. The first wiring condition is described mathematically by the rule: $i \rightarrow j$ if $\alpha\ exp\left(-\ \delta_{ij}/\beta\ l\right) < p_o$, where i and j is points in the dataset, δ_{ij} is the Euclidean distance between i and j, l is a reference length, α and β are model parameters, and p_o is a threshold. In the equation, the symbol → stands for connect. This rule is a variation of the celebrated Waxman algorithm (*29*). To implement the second rule, one has to determine, for each node i of the dataset, the function $\zeta(i) = e^{-(\gamma_{max}/\gamma-1)}$, where $\gamma = \rho\ d_{min}$, and ρ and d_{min} are the local density of i (ρ) and the minimum distance to other points with higher density than i (d_{min}), and γ_{max} is the maximum value of γ calculated over the full dataset. High values of ζ are indicative of cluster centers. Then, two nodes i and j are connected if $\zeta(i) > p_d$ and $\zeta(j) > p_d$, where p_d is a threshold. This rule, developed by us, was inspired by the fundamental work of Rodriguez and Laio (*30*). The combination of rules generates both short and long range connections. In this study, the parameters of the model were fixed as $\alpha = 1$, $\beta = 0.025$, $p_d = 0.9$, while p_o was uniformly varied in the $0.84 - 0.96$ interval.

Supplementary Note 15. *Simulating information flows in neuronal cell networks.*

Networks of neuronal cells built using the methods described above were stimulated with an external disturbance to understand how a different network topology on soft PDMS surfaces affects cell-signaling. Upon application of the external stimulus I_{stim}, the potential V measured across the membrane of the targeted neuron varies with time, the variation being described by the differential equation of time (t) (*31*, *32*):

$$C_m \frac{dV}{dt} = -g_l(V - V_o) + I_{stim}, \quad (14.1)$$

where $C_m = 300\,pF$ is the capacitance of the membrane, $g_l = C_m/\tau$ is the conductance of the neuron, $\tau = 3\,ms$ is the time constant, $V_o = 6\,mV$ is the resting potential. Moreover, the stimulus I_{stim} is modelled as a discrete-binary sequence of $0/J$ values. J is a current pulse such that $J/C_m = 0.25\,mV$. The number of pulses in a sequence (w) and the repetition of sequences in the train has been chosen being $w = 8$ and $N = 50$. Notice that equation (14.1) is the celebrated leaky integrate and fire model (*33*, *34*), the solution of which (V) is the potential at the postsynaptic sites of the neuron as a function of time. V is then compared to a reference $(V_{th} = 9\,mV)$ that, when exceeded $(V > V_{th})$, triggers the discharge of an action potential (AP). Action potentials generated over time in the originating neuron (o) are then commuted as an input to the neurons i connected to o through links of the network, $J \sum_k^{APN} \delta\left(t - t_i^k\right)$. Where δ (the Kronecker delta) is $\delta = 1$ if $t = t_i^k$ and $\delta = 0$ otherwise. Moreover: t is the absolute time, t_i^k is the time pattern of AP events occurring in o, k is an index running from 1 to the last discretized time of APs (APN). This is the mechanism by which an initial disturbance is transported in the network. As a result, neurons of the network produce over time spike trains conveniently converted in $0/1$ discretized sequences of bits for subsequent storage and analysis.

Supplementary Note 16. *Functional multi calcium imaging.*

Soft PDMS substrates were individually placed in 12-multi-well plates (Corning Incorporated) and sterilized under UV irradiation for 12 h. Poly-D-lysine (PDL) (Sigma-Aldrich P6407) was diluted in sterile H2O to a final concentration of 0.1 mg/ml and used to cover the substrates prior neuronal cells culture. Substrates were left in the PDL solution overnight in a cell culture incubator (37 °C, 5% CO2, 90% humidity). Neuronal cells were extracted from Sprague Dawley rat embryos brains at day 18 (E18) as described in previous works (*35*, *36*). All procedures were carried out in accordance with the guidelines established by the European Communities Council (Directive of November 24th, 1986) and approved by the National Council on Health and Animal Care (authorization ID 227, prot. 4127, 25th March 2008). Pregnant females were deeply anesthetized with CO2 and decapitated embryos (embryonic day E18) were removed and decapitated; brains were removed from the skulls and put in cold HBSS (Thermo Fisher Scientific 14175129); the hemispheres were divided and the meninges removed: the hippocampi were dissected out and incubated in 5 ml trypsin-EDTA 0.25% (Thermo Fisher Scientific 25200056) in water bath at 37° for 30 minutes. After incubation were add to trypsin solution a few ml of Neurobasal (Thermo Fisher Scientific 21103049) + 1% Pen Strep (Thermo Fisher Scientific 15140122) + 1% Glutamax (Thermo Fisher Scientific 35050038) + 2% B27 (Thermo Fisher Scientific 17504044) + 10% Fetal Bovine Serum (Thermo Fisher Scientific 10270106); cell suspension was centrifuged for 5 min at 1200 RPM. The supernatant was discarded and added fresh Neurobasal + 10% FBS. Then the hippocampi were dissociated by gently pipetting; the solution was filtered with a cell strainer (40um pore size) and centrifuged 7 min at 700 RPM; pellet of cells was diluted at the desired density (1000 cell/ul) in complete Neurobasal and plated on surfaces. Neurons were incubated at 37 °C in a humidified 5% CO2/air atmosphere. Then, PDMS surfaces with varying compliance, hosting neuronal cells in networks, were incubated in 1 ml of dye solution (Fluo4, Life Technologies/Thermo Fisher, at a 1:2000 dilution) at room temperature for 20 min in dark conditions. Samples were then washed once with PBS and immediately imaged over time with a fluorescent upright microscope (Leica DM6000, Wetzlar, Germany), using the following parameters: Exposure time=30 ms; Frame rate=300 ms. The overall images were acquired with the LAS AF software (Leica Microsystems Srl) (*35*, *36*).

Supplementary References

(*1*) L. Bruno, S. Canto, L. Luciani; Localized heat treatment to improve the formability of steel pipes for hydraulic applications: process design and mechanical characterization; International Journal of Advanced Manufacturing Technology (2022), 119 (1-2), 927-940; DOI: 10.1007/s00170-021-08321-7

(*2*) W. Oliver, G. Pharr; Measurement of hardness and elastic modulus by instrumented indentation: Advances in understanding and refinements to methodology; Journal of Materials Research (2004), 19(1), 3-20; DOI: 10.1557/jmr.2004.19.1.3

(*3*) Metallic materials – Instrumented indentation test for hardness and materials parameters; ISO 14577-1; International Standards Office; Geneva; 2015.

(*4*) Standard Practice for Instrumented Indentation Testing; ASTM E2546-15; ASTM International; Wst Conshohocken; 2015.

(*5*) N.K. Mukhopadhyay, P. Paufler; Micro- and nanoindentation techniques for mechanical characterisation of materials; International Materials Reviews (2006), 51(4), 209-245; DOI: 10.1179/174328006X102475.

(*6*) J. Antons, M.G.M. Marascio, J. Nohava, R. Martin, L.A. Applegate, P.E. Bourban, D.P. Pioletti; Zone-dependent mechanical properties of human articular cartilage obtained by indentation measurements; Journal of Materials Science: Materials in Medicine (2018), 29(51), art.n. 57; DOI: 10.1007/s10856-018-6066-0.

(*7*) S. V. Kontomaris and A. Malamou. Hertz model or Oliver & Pharr analysis? Tutorial regarding AFM nanoindentation experiments on biological samples. Materials Research Express 7(3): 033001. 2020.

(*8*) Dichu Xu, Terence Harvey, Eider Begiristain, Cristina Domínguez, Laura Sánchez-Abella, Martin Browne, Richard B. Cook. Measuring the elastic modulus of soft biomaterials using nanoindentation. Journal of the Mechanical Behavior of Biomedical Materials 133: 105329. 2022.

(*9*) D Di Mascolo, A Coclite, F Gentile and M Francardi. Quantitative micro-Raman analysis of micro-particles in drug delivery. Nanoscale Advances, 2019, 1, 1541-1552.

(*10*) Angel S. Cruz-Felix, Agustin Santiago-Alvarado, Josimar Marquez-García, Jorge Gonzalez-García. PDMS samples characterization with variations of synthesis parameters for tunable optics applications. Heliyon 5: e03064, 2019.

(*11*) A Zahid, B Dai, R Hong and D Zhang. Optical properties study of silicone polymer PDMS substrate surfaces modified by plasma treatment. Mater. Res. Express 4: 105301. 2017.

(*12*) Alex Rodriguez and Alessandro Laio, Clustering by fast search and find of density peaks, Science 322(6191): 1492-1496, 2014.

(*13*) Danielle Carrier, Michel Pezolet. Raman spectroscopic study of the interaction of poly-L-lysine with dipalmitoylphosphatidylglycerol bilayers. Biophysical Journal 46(4): 497–506, 1984.

(*14*) Julian F. A. Perlitz, Lukas Gentner, Phillipp A. B. Braeuer and Stefan Will. Measurement of Secondary Structure Changes in Poly-L-lysine and Lysozyme during Acoustically Levitated Single Droplet Drying Experiments by In Situ Raman Spectroscopy. Sensors 22: 1111, 2022.

(*15*) Fanse S, Bao Q, Zou Y, Wang Y, Burgess DJ. Effect of crosslinking on the physicochemical properties of polydimethylsiloxane-based levonorgestrel intrauterine systems. Int J Pharm. 2021 Nov 20;609:121192. doi: 10.1016/j.ijpharm.2021.121192. Epub 2021 Oct 16. PMID: 34666142; PMCID: PMC9236551.

(*16*) Regehr KJ, Domenech M, Koepsel JT, Carver KC, Ellison-Zelski SJ, Murphy WL, Schuler LA, Alarid ET, Beebe DJ. Biological implications of polydimethylsiloxane-based microfluidic cell culture. Lab Chip. 2009 Aug 7;9(15):2132-9. doi: 10.1039/b903043c. Epub 2009 Jun 4. PMID: 19606288; PMCID: PMC2792742.

(*17*) Sarah-Sophia D. Carter, Abdul-Raouf Atif, Sandeep Kadekar, Ingela Lanekoff, Håkan Engqvist, Oommen P. Varghese, Maria Tenje, Gemma Mestres, PDMS leaching and its implications for on-chip studies focusing on bone regeneration applications, Organs-on-a-Chip, Volume 2, 2020, 100004, ISSN 2666-1020,https://doi.org/10.1016/j.ooc.2020.100004.

(*18*) Sivakumar Murugadoss, Dominique Lison, Lode Godderis, Sybille Van Den Brule, Jan Mast, Frederic Brassinne, Noham Sebaihi, Peter H Hoet. Toxicology of silica nanoparticles: an update. Arch Toxicol. 91(9):2967-3010. 2017.

(*19*) Hamsa Jaganathana and Biana Godin. Biocompatibility Assessment of Si-based Nano- and Microparticles. Adv Drug Deliv Rev. 64(15): 1800–1819. 2012.

(*20*) Decuzzi, P. & Ferrari, M. Modulating cellular adhesion through nanotopography. Biomaterials 31, 173–179 (2010).

(*21*) Sackmann, E. & Smith, A.-S. Physics of cell adhesion: some lessons from cell mimetic systems. Soft Matter 10, 1644–1659 (2014).

(*22*) Borodich, F. M. in Advances in Applied Mechanics Vol. 47 (eds Daniel Balint & Stephane Bordas) 225-366 (Academic Press, 2014).

(*23*) Ciavarella, M., Joe, J., Papangelo, A. & Barber, J. R. The role of adhesion in contact mechanics. Journal of the Royal Society Interface 16, 20180738 (2019).

(*24*) Gentile, F. Cell aggregation on rough surfaces. Journal of Biomechanics 115, 110134 (2021).

(*25*) Marinaro, G. *et al.* Networks of Neuroblastoma Cells on Porous Silicon Substrates Reveal a Small World Topology. *Integrative Biology* 7, 184-197 (2015).

(*26*) Steinley, D. & Brusco, M. J. Initializing K-means Batch Clustering: A Critical Evaluation of Several Techniques. *Journal of Classification* 24, 99–121 (2007).

(*27*) Aprile, F., Onesto, V. & Gentile, F. The small world coefficient 4.8±1 optimizes information processing in 2D neuronal networks. *npj Systems Biology and Applications* 8, 1-11 (2022).

(*28*) Gentile, F. Multipoint connection by long-range density interaction and short-range distance rule. *Physica Scripta* 96, 045004 (2021).

(*29*) Waxman, B. Routing of multipoint connections. *IEEE Journal on Selected Areas in Communications* 6, 1617–1622 (1988).

(*30*) Rodriguez, A. & Laio, A. Clustering by fast search and find of density peaks. *Science* 344, 1492–1496 (2014).

(*31*) Aprile, F., Onesto, V. & Gentile, F. The small world coefficient 4.8±1 optimizes information processing in 2D neuronal networks. *npj Systems Biology and Applications* 8, 1-11 (2022).

(*32*) Onesto, V. *et al.* Information in a Network of Neuronal Cells: Effect of Cell Density and Short-Term Depression. *BioMed Research International* 2016, 1-12 (2016).

(*33*) de la Rocha, J. & Parga, N. Short-Term Synaptic Depression Causes a Non-Monotonic Response to Correlated Stimuli. *The Journal of Neuroscience* 25, 8416–8431 (2005).

(*34*) FitzHugh, R. Mathematical models of threshold phenomena in the nerve membrane. *Bulletin of Mathematical Biology* 17, 257–278 (1955).

(*35*) Onesto, V.; Cancedda, L.; Coluccio, M.; Nanni, M.; Pesce, M.; Malara, N.; Cesarelli, M.; Fabrizio, E. D.; Amato, F.; Gentile, F. *Scientific Reports* 2017, 7, (9841), 1-13.

(*36*) Onesto, V.; Villani, M.; Narducci, R.; Malara, N.; Imbrogno, A.; Allione, M.; Costa, N.; Coppedè, N.; Zappettini, A.; Cannistraci, C. V.; Cancedda, L.; Amato, F.; Di Fabrizio, E.; Gentile, F. *Scientific Reports* 2019, 9, 4021.
